# Measuring Competitiveness at NUTS3 Level and Territorial Partitioning of the Italian Provinces

**DOI:** 10.1007/s11205-021-02836-y

**Published:** 2022-10-06

**Authors:** Pierpaolo D’Urso, Livia De Giovanni, Francesca G. M. Sica, Vincenzina Vitale

**Affiliations:** 1 Department of Social Sciences and Economics, Sapienza University of Rome, Rome, Italy; 2 Department of Political Sciences, Luiss university and Data Lab Luiss, Rome, Italy; 3 Economic Research Department, Confindustria and Data Lab Luiss, Rome, Italy

**Keywords:** Competitiveness, Territorial attractiveness, NUTS3, Spatial constraints, Fuzzy partitioning around medoids

## Abstract

In this paper we propose a dashboard of indicators of territorial attractiveness at NUTS3 level in the framework of the EU Regional Competitiveness Index (RCI). Then, the Fuzzy C-Medoids Clustering model with multivariate data and *contiguity* constraints is applied for partitioning the Italian provinces (NUTS3). The novelty is the territorial level analized, and the identification of the elementary indicators at the basis of the construction of the eleven composite competitiveness pillars. The positioning of the Italian provinces is deeply analyzed. The clusters obtained with and without contraints are compared. The obtained partition may play an important role in the design of policies at the NUTS3 level, a route already considered by the Italian government. The analysis developed and the related set of indicators at NUTS3 level constitute an information base that could be effectively used for the implementation of the National Recovery and Resilience Plan (NRRP).

## Introduction

The term “territorial attractiveness” is a binomial shared by economists and economic geographers to identify a series of assets with which the territories are equipped. The intensity of individual assets and a favorable combination of different assets can represent an attractive factor to direct preferences towards a given territory rather than another for residential and productive settlements, respectively of private citizens (residential attractiveness) of foreign and national investors (productive attractiveness). Less universally accepted is the use, or rather the abuse of the concept of territorial competitiveness. Unlike the concepts of “utility” and “efficiency”, competitiveness is not a basic construct in economics and analyses of competitiveness have in general no fundamentals that are strictly anchored to economic theory. From a macroeconomic point of view, various official definitions of territorial (country) competitiveness can be found featuring at least one of the following elements: economic performance, in terms of productivity growth rate and real income; international trade in goods and services; sustainability, understood as long-term sustainable achievements. In the European Competitiveness Report (2000) we find the following: “An economy is competitive if its population can enjoy high and rising standards of living and high employment on a sustainable basis. More precisely, the level of economic activity should not cause an unsustainable external balance of the economy nor should it compromise the welfare of future generations”. If at the sectoral level the adaptation of the concept does not present any problems whatsoever, at the macroeconomic level some conceptual dyscrasias arise. The basic idea of the supporters of extending the micro concept of corporate competitiveness to the whole country is that this can be considered as the sum of the companies that operate there, or as a single large company that is operating on international markets with an ever increasing number of competitors (Porter 2004; Rucinska and Rucinsky 2007). It is precisely because of the similarity between company and country that economists consider the translation of the concept from the micro to the macro level as unacceptable (Krugman 1994). On a closer inspection, the implicit analogy between business and territory is for many economists meaningless, as competition between countries cannot, for obvious reasons, lead to the expulsion or suppression of the less competitive ones (Krugman 1994). On the contrary, the success of a territory (like a country or a region within a country) may in general benefit its neighboring territories thanks to the effects of positive spillovers. In essence, the competitive game between countries is not zero-sum, but rather a plus-sum game. The success of a country or region creates more than destroys the opportunities for others and as known, trade among nations is not a game “without results” (Psofogiorgos and Metaxas 2016).^1^ The concept of regional competitiveness, adopted by the European Commission (EC) when drawing up the Regional Competitiveness Index (RCI, from now on), lies somewhere between the microeconomic concept (firm) and the macroeconomic one (country). “Regional competitiveness can be defined as the ability to offer an attractive and sustainable environment for firms and residents to live and work.” If, therefore, competitiveness is the ability to offer an attractive environment, then the two concepts of competitiveness and attractiveness end up merging into one another (Davies et al. 2000).

The measures of attractiveness proposed here, the “pillars”, represent dimensions or aspects of attratctiveness. Each pillar is obtained through techniques of multivariate statistical analysis as the synthesis of a plurality of indicators, so that both the causes, input, and the effects, outcome, of attractiveness in the territory are captured transversally. The comparative evaluation makes it possible to carry out a precise anamnesis of the territory through the “components” of the pillars and then to define the “cure” with the formulation of policy proposals tailored to each territory. The methodological approach for the construction of the pillars is not new, but has been borrowed from the Regional Competitiveness Index (RCI) of the European Commission. The originality of the work consists in the lower territorial level, that has influenced the choice of indicators within each pillar.

Unfortunately, the information available at the territorial level provided by official statistics is published in different databases depending on the topic and is therefore dispersed in many information sources. And yet they are fundamental for an exhaustive and in-depth reading of local specificities. Local specificities are preparatory to the formulation of local policies aimed at raising the potential attractiveness.

The clustering procedure adopted enjoys the benefits connected to Fuzzy clustering and to Partitioning Around Medoids (PAM). Due to the difficulty in identifying a clear boundary between clusters in real applications involving territorial units, i.e. provinces even belonging to the same region, fuzzy clustering is more attractive than the hard clustering methods (D’Urso 2014. The PAM approach allows for more appealing and easy to interpret results of the final partition (Kaufman and Rousseeuw 2005), determining real and not virtual representatives of the clusters.

In this paper we propose a dashboard of indicators of territorial attractiveness at NUTS3 level in the framework of the EU Regional Competitiveness Index (RCI). Then, the Fuzzy C-Medoids Clustering model with multivariate data and *contiguity* constraints is applied for partitioning the Italian provinces (NUTS3). The novelty is the territorial level analized, and the identification of the elementary indicators at the basis of the construction of the eleven composite competitiveness pillars.

The paper is structured as follows. In Sect. 2 the competitiveness indicators at NUTS3 level and related pre-processing are presented. In Sect. 3 the clustering model is introduced. In Sect. 4 the model is used for clustering the Italian provinces. Section 5 presents the Conclusions.

## Indicator of Competiveness at NUTS3 Level (Provinces)

The Regional Competitiveness Index (RCI) (Annoni and Dijkstra 2019) is composed of eleven pillars that describe the different aspects of competitiveness. They are classified into three groups (subindexes): *Basic*, *Efficiency* and *Innovation*.

The Basic group includes five pillars: (1) Institutions; (2) Macroeconomic Stability; (3) Infrastructure; (4) Health; (5) Basic Education. These represent the key basic drivers of all types of economies.

The Efficiency group includes three pillars: (6) Higher Education; (7) Labor Market Efficiency; (8) Market Size.

The Innovation group includes three pillars: (9) Technological Readiness; (10) Business Sophistication; (11) Innovation.

The pillars are composite variables. The complete list of all candidate indicators at the NUTS2 level can be found in The EU Regional Competitiveness index 2019 (Annoni and Dijkstra 2019). The partition of the European regions (NUTS2) with respect to the Basic, Efficiency and Innovation subindexes has been analized in D’Urso et al. (2019b).

In the data warahouse of the National Institute of Statistics (Istat) there is no theme specifically dedicated to the territory but it is possible to download from each macro theme the territorial detail through the customization options of the default layout and analyze the phenomena of interest from a triple perspective:

- Spatial: to analyze the relative positioning of the territories (regions and provinces);
- Temporal: to grasp the evolution of a given phenomenon over time at a national and territorial level (region or province);
- Sectoral: to analyze productive specialization and its territorial articulation.

For this reason, the collection of quantitative territorial data at the provincial level (“NUTS3” European glossary, “Small regions” OECD glossary) was the most challenging phase of this analysis due to the difficulty of finding updated and transversal data on the various themes of interest in a single source of information. Thanks to the fusion of a number of official national (Istat, Unioncamere, Bank of Italy, Cnel) and international (Eurostat, OECD) information sources, the number of variables collected was quite large, but the creation of a complete territorial database required careful prior selection based on the criterion of relevance to the eleven dimensions chosen to describe the phenomenon of attractiveness. In the end, over 150 indicators were selected for each territorial unit and catalogued in each pillar. This second phase of systematization of the data collected was easier because it was possible to move along a path already traced and regularly updated in scientific work in Europe. The selection of the elementary indicators and their subsequent cataloguing within the pillars was inspired, in fact, by the methodology published in the reports of the European Commission to calculate the RCI (Annoni and Dijkstra 2019) and of the Word Economic Forum to calculate the Global Competitiveness Index. The originality of this study is twofold and consists, on the one hand, in having replicated at the NUTS3 provincial level the measurement approach now consolidated at the regional level (NUTS2) and, on the other, in having included exclusively indicators referring to the provinces. It must be said that this has been made possible by the Istat initiative to elaborate Equitable and Sustainable Well-being not only at the national level but also at the level of the territories (BES of the territories) thanks to which a rich set of indicators for each of the twelve domains in which the BES has been articulated has been made available to the government and citizens with coverage of all 110 provincial administrative units.

In the paper, to obtain the pillars, the RCI methodology is used.

Firstly the indicators describing each of the eleven attractiveness aspects for the Italian provinces are identified. To correct for different range and measurement units, weighted z-scores are adopted using the provinces’ population sizes as weights. The Principal Component Analysis (PCA) is used to select the indicators within each pillar. Then the eleven pillars are computed as a simple average of the selected indicators in each pillar, and next the subindexes Basic, Efficiency, Innovation are computed as a simple average of the pillars in each subindex. The use of simple averages in the two steps is based on the Principal Component Analysis, used to check for the internal consistency of the indicators within each pillar and to determine the sign (positive or negative) of the indicators. The conditions to be verified to use only one pillar - obtained as a simple average of the indicators measuring that pillar - are that each pillar shows a unique, most relevant principal component accounting for a large amount of variance and that all the indicators contribute to approximatively the same extent to the first principal component.

The sources utilized are institutes of official statistics with the exception of “Fondazione Etica su dati Amministrazione Trasparente”.^2^

The selected indicators in the pillars of the Basic group are presented in Table 1.

**Table 1:** Indicators of the subindex Basic

Subindex	Pillar	Indicator (source, year)
Basic	Institutions	Pending trials (2016) (reversed) (Ministry of Justice, DG-STAT)
Basic	Institutions	Trial duration (reversed) (2016) (Ministry of Justice, DG-STAT)
Basic	Institutions	Vote participation (BES-Istat, average 2004, 2009, 2014, 2019)
Basic	Institutions	Female municipal administrators out of total local administrators (BES-Istat, 2018)
Basic	Institutions	Social relations intensity: Non profit organizations, per 10000 population (BES-Istat, 2017)
Basic	Institutions	Administrative capacity (NUTS3 level) (Fondazione Etica su dati Amministrazione Trasparente, 2020)
Basic	Institutions	Corruption Last 3 years (I.stat-Justice and Security, 2015–2016))
Basic	Institutions	Bribe Health (I.stat-Justice and Security, 2015–2016)
Basic	Institutions	Bribe Assistance (I.stat-Justice and Security, 2015–2016)
Basic	Institutions	Bribe Education (I.stat-Justice and Security, 2015–2016)
Basic	Institutions	Bribe Job (I.stat-Justice and Security, 2015–2016)
Basic	Institutions	Bribe administration (I.stat-Justice and Security, 2015–2016)
Basic	Macroeconomic stability	Surplus (deficit) of administration in relation to current revenues (I.stat Public Adm. and Private Inst., 2017)
Basic	Macroeconomic stability	Collection capacity (BES-Istat, 2017)
Basic	Macroeconomic stability	Interest expenses in relation to current revenues (reversed) (I.stat Public Adm. and Private Inst., 2017)
Basic	Infrastructure	Accessibility (travel times) index towards urban and logistic nodes (reversed) (Istat Indicators for Development, 2013)
Basic	Infrastructure	Seats km offered by all modes of transport per inhabitant (BES-Istat, 2018)
Basic	Infrastructure	Annual passenger density in local public transport and airports per inhabitant (BES-Istat,, 2017)
Basic	Infrastructure	Car-sharing: availability of vehicles per 100 thousand inhabitants (Istat-Urban environment, 2017)
Basic	Health	Life expectancy at birth, average number of years (BES-Istat, 2018)
Basic	Health	Infant mortality per 1.000 live births (BES-Istat, 2017)
Basic	Health	Cancer mortality (20–64 years) - standardized rates per 10.000 residents (reversed) (BES-Istat, 2017)
Basic	Health	Hospital outmigration to other region for ordinary acute hospitalizations (BES-Istat, 2018) (reversed)
Basic	Basic education	Vocational (vocational) graduates: technical and vocational graduates (Eurostat), 2018
Basic	Basic education	Students’ reading proficiency level - mean score (OECD - PISA, 2018)
Basic	Basic education	Students’ numeracy proficiency level - mean score (OECD - PISA, 2018)
Basic	Basic education	Underachievement rate in reading (reversed) (Invalsi, 2019)
Basic	Basic education	Underachievement rate in numeracy (reversed) Invalsi, 2019)

*Pillar I - Institutions* Recognition of the role of institutions in shaping a country’s ”fate” has gained relevance as a result of a new strand of research that identifies institutions as another cause of differentials in the development rates of economies in addition to traditional factors (Acemoglu et al. 2001). The empirical literature has emphasized the links between institutional soundness and the following aspects of an economic system: resolution of market failures and improved efficiency (Streeck and Schmitter 1991); reduction in transaction costs (North 1990); stimulation of innovation and productivity (Putnam 2000).

What are Institutions? According to Douglass North (North 1990): “are the rules of the game in a society or, more formally, are the humanly devised constraints that shape human interaction”. Two important characteristics emerge from the definition: 1. the human component (“humanly devised”) that overlaps with other factors such as natural geographic factors that are beyond human control; 2. constraints on human behavior (“the rules of the game” setting “constraints” on human behavior). Candidate indicators to measure the “institutions” dimension must be able to capture the quality and efficiency of institutions and the regulatory environment that impacts on the ease of “doing business”. Other indicators capture the phenomenon of corruption through an *ad hoc* module included by Istat in the 2015-2016 Citizens’ Security Survey (NUTS3 level).

*Pillar II - Macroeconomic stability* A situation of sound finances at the local level is essential for the public operator to receive confidence in its solvency from private operators, whether they are consumers or producers of goods and services. The risk of financial imbalances impacts on confidence which is, in turn, crucial to raising the rate of investment in the long term, a fundamental ingredient for preserving the competitiveness of an area.

*Pillar III - Infrastructure* The fourth industrial revolution is making possible, thanks to digital technology, a closer connection between production systems located in different places. This paradigm shift influences the competitiveness factors of the territories by making logistics enter the top ten of the winning elements, not only as storage and sorting, but increasingly as an ancillary and accessory service to production and as an advanced service with high technological content. Modern and functioning infrastructures contribute in fact to increase the economic efficiency and the social equity through the maximization of local economic potential (Rodriguez-Pose and Crescenzi 2008). In addition, they promote accessibility to other regions and countries, contributing to the integration of peripheral areas. Others authors (Lopez-Claros et al. 2007) emphasize the key role of infrastructure in determining the location of economic activities and in influencing the development of certain types of productive activities. The impact on the competitiveness of territories is conveyed by the increase in economic efficiency.

*Pillar IV - Health* Health is a crucial dimension for the well-being of the citizens who reside in a territory and for this reason an *ad hoc* pillar is dedicated to it that describes the health conditions of the population. A healthy workforce is a key factor for the increase of the rate of activity in the labor market and for the increase in labor productivity at the regional and national levels (Official Journal of the European Union). Of course, the link with competitiveness is indirect in that mediated by the impact of healthy living conditions.

*Pillar V - Basic Education* Unlike the availability of natural resources, the endowment of human capital of an area, is not fixed but can be increased by investing in education which, in turn, produces a return that from the private point of view proves to be higher than other forms of investment available to households, who must decide how to allocate their financial capital between alternative investments (Coleman 1988). There are a number of empirical studies demonstrating the existence of a positive association between educational quality and economic growth (Hanushek and Woessmann 2007). International tests of learning outcomes from primary school to adults at work aim to capture the quality of the human capital compared to quantitative measures. There are also empirical evidences that adult competences applied at work enhance labor productivity at company level and activate the virtuous circle from human capital to a strong, sustainable and balanced growth by disseminating new technologies and work-organization practices. The transition from a traditional knowledge-based to a competence-based educational-training system is by now unavoidable. The quality of education is measured by the results obtained in cognitive tests, whose purpose is to assess not only “knowledge” but also theoretical knowledge. The most widely used test for measuring skills is PISA, which stands for Programme for International Student Assessment, an OECD initiative that, scheduled every three years, measures the reading, mathematics and science skills of 15-year-old students.

The selected indicators in the pillars of the Efficiency group are presented in Table 2.

**Table 2:** Indicators of the subindex Efficiency

Subindex	Pillar	Indicator (source, year)
Efficiency	Higher education	Percentage incidence of tertiary graduates 25–39 (BES-Istat, 2019)
Efficiency	Higher education	Transition to tertiary education (BES-Istat, 2017)
Efficiency	Higher education	Life Long Learning (I.stat, 2018)
Efficiency	Higher education	Early school leavers (BES-Istat, 2017)
Efficiency	Higher education	Stem graduates (author elaboration on I.stat, 2018)
Efficiency	Labor market efficiency	Employment rate 15–64 years (I.stat, 2019)
Efficiency	Labor market efficiency	Gender Gap - employment rate (author elaboration on I.stat, 2019)
Efficiency	Labor market efficiency	Missing work participation rate (BES-Istat, 2019) (reversed)
Efficiency	Labor market efficiency	Gender Gap - missing work participation (author elaboration on BES-Istat, 2019) (reversed)
Efficiency	Labor market efficiency	Share 15–24 not in education, employment, training (NEET) (BES-Istat, 2019) (reversed)
Efficiency	Labor market efficiency	Labor productivity (author elaboration on I.stat, 2017)
Efficiency	Labor market efficiency	Formal Job (reversed) (BES-Istat, 2018)
Efficiency	Labor market efficiency	Fatal accidents at work (reversed) (BES-Istat, 2017)
Efficiency	Labor market efficiency	Wages of tertiary graduates (I.stat, 2017)
Efficiency	Market size	Provincial GDP year 2017-Constant prices base year 2015 (I.stat, 2017)
Efficiency	Market size	Population (I.stat, 2020)
Efficiency	Market size	Distance of 2017 GDP from pre-crisis GDP levels - index numbers 2007=100 (author elaboration on I.stat, 2017)
Efficiency	Market size	Potential market in terms of GDP provincial incidence on Italy GDP (author elaboration on I.stat, 2017)
Efficiency	Market size	Propensity to export (author elaboration on I.stat, 2017)
Efficiency	Market size	Propensity to import (author elaboration on I.stat, 2017)
Efficiency	Market size	Non-performing loans loans (BES-Istat, 2019) (reversed)

*Pillar VI - Higher Education* The contribution of education to productivity and growth has been extensively studied. Knowledge and innovation-based economies need well-educated, adaptable human capital and an education system capable of transmitting not only theoretical knowledge but also practical skills and, hence, competencies. In a context increasingly permeated by knowledge, universities and businesses play a decisive role: the former because they are typically the places where knowledge is cultivated, accumulated and transmitted; the latter because they have the task of applying the results of research to production techniques, products and business organization.

*Pillar VII - Labor Market Efficiency* An efficient and flexible labor market favors an optimal allocation of resources (Lopez-Claros et al. 2007) which is reflected in the attractiveness of an area that is a precondition for its competitiveness understood as competition that is triggered between territories in order to catalyze the preferences of potential “users” of the area, as investors (new or existing) who must evaluate the best location for their production facilities, but also as citizens who must decide where to live. Employment and unemployment rates provide information on the level of activity in the local labor market, while a long-term unemployment rate is a symptom of the existence of structural problems. The differential in employment rates between women and men is an important aspect and signals a lack of reconciliation between work and family life, the burden of which falls on women who are often forced to leave the labor market and swell the ranks of the inactive.

*Pillar VIII - Market size* The pillar describes the potential outlet market available to firms: the larger the market, the greater the possibility of exploiting economies of scale and benefiting from the gains from them in terms of reduced fixed costs. Market size encourages entrepreneurship and fosters innovation. The problem is not so much the availability of a large market but rather the accessibility to it. The potential of the market is captured in terms of absolute values of population, Gross Domestic Product and spending capacity.

The selected indicators in the pillars of the Innovation group are presented in Table 3.

**Table 3:** Indicators of the subindex Innovation

Subindex	Pillar	Indicator (source, year)
Innovation	Technological readiness	Ultrabroadband penetration (Indicators for Development-Istat, 2017)
Innovation	Technological readiness	Number of firms registered in the innovative SME section (Ministry of Economic Development, 2019)
Innovation	Technological readiness	Manufacturing specialization in high-tech sectors (A misura di Comune-Istat, 2015)
Innovation	Technological readiness	Active enterprises with 3 and more employees engaged in Innovation projects (Companies Census-Istat, 2018)
Innovation	Technological readiness	Active enterprises with 3 and more employees using digital platforms (Companies Census-Istat, 2018)
Innovation	Technological readiness	Number of online services made available to citizens by the local PA (Istat, 2018)
Innovation	Business sophistication	Business fragmentation: percentage share of micro, small and medium-sized enterprises (ASIA-Istat, 2018)
Innovation	Business sophistication	Agriculture, forestry and fishing specialization index - value added (I.stat National Accounts, 2017) (reversed)
Innovation	Business sophistication	Industry specialization index - value-added (I.stat National Accounts, 2017)
Innovation	Business sophistication	Construction specialization index - value added (I.stat National Accounts, 2017) (reversed)
Innovation	Business sophistication	Services specialization index - value added (I.stat National Accounts, 2017)(reversed)
Innovation	Business sophistication	Entrepreneurship intensity per thousand inhabitants (I.stat, 2018)
Innovation	Business sophistication	Number of total businesses registered in the cultural production system (ASIA-Istat, 2018)
Innovation	Business sophistication	Degree of openness to foreign trade (author elaboration on I.stat, 2017)
Innovation	Innovation	Propensity to patent - applications filled at the European Patent Office (EPO) (UIBM database, 2016)
Innovation	Innovation	Propensity to patent - number of patents applications to the Italian Patent Office (UIBM database, 2018)
Innovation	Innovation	Registered patents to the Italian Patent Office (UIBM database, 2018)
Innovation	Innovation	Registered trademarks by province of registration in Italy (UIBM database, 2018)
Innovation	Innovation	Brain gain/drain or mobility of Italian graduates (25–39 years) (BES-Istat, 2017)
Innovation	Innovation	Cultural business employees as a percentage of total active business employees (ASIA-Istat, 2017)

*Pillar IX - Technological Readiness* This dimension captures the degree to which households and businesses are using ICT technologies. The Fourth Industrial Revolution is changing the way we produce under the banner of the three “v’s”: volume, velocity, variety. Increasingly high production volumes, greater speed in the production of goods and services and, finally, wider variety of products. Compared to previous revolutions, with digital technology both the time lapse between discovery, application and diffusion of innovations and the distance between things, people and countries have become much shorter thanks to connectivity. The way in which new information and communication technologies are used by a firm’s workers depends closely on the degree of penetration and diffusion of these technologies in everyday life. Empirical evidence shows how the adoption and diffusion of ERP (Enterprise Resource Planning) and CRM (Customer Relationship Management) applications is strongly dependent on the size of the firm, but a crucial role is played by the level of education of employees rather than of the entrepreneur.

*Pillar X - Business Sophistication* The degree of maturity of the productive system provides an indication of the level of productivity achieved by the area in response to competitive pressure from other areas, including those beyond its borders. Specialization in sectors with high added value, such as industry, contributes to raising territorial competitiveness.

*Pillar XI - Innovation* Innovation is the true engine of growth. More than costs, more than the availability of raw materials, more than geographical location, innovation is the key factor in the competitiveness of a country and a territory, especially the developed ones, as underlined by Lopez-Claros et al. (2007). In its annual report, the World Bank highlights the positive correlation between knowledge and growth and underlines how the fastest growing economies are also those with a higher Knowledge Economy Index (KEI). Unlike developing areas, where it is the increase in domestic consumption induced by the rise in the standard of living that drives GDP growth, in mature economies growth is fueled by technological innovation that stimulates the replacement of existing goods through the creation of new or higher performance goods: the faster the replacement of goods, the higher the growth rate. For innovation to spread throughout the territorial economy, the institutional environment must be sufficiently pervasive to create collaborative relationships between knowledge infrastructures (universities and research centers) and the firms that must apply the results of innovation to processes and products (Cantwell 2006). Empirical research shows that knowledge production is quite concentrated (Audretsch and Feldman 1996), so innovative firms tend to locate in settings with specialized human capital, which in turn tends to accumulate further in areas that are vibrant in terms of innovation.

For detailed description of the indicators for each pillar see the Sect. 5 (Appendix).

The values of the subindexes Basic, Efficiency and Innovation for the 106 regions are presented in Table 4.

**Table 4:** Basic, Efficiency, Innovation by province

Region	Province	B	E	I	Region	Province	B	E	I
Piemonte	Torino	0.46	0.62	0.75	Toscana	Lucca	−0.09	-0.08	-0.11
Piemonte	Vercelli	− 0.01	− 0.09	− 0.27	Toscana	Pistoia	0.20	− 0.21	− 0.34
Piemonte	Novara	0.05	0.20	0.20	Toscana	Firenze	0.52	0.65	0.55
Piemonte	Cuneo	0.30	0.12	− 0.13	Toscana	Livorno	− 0.01	− 0.19	− 0.13
Piemonte	Asti	0.26	− 0.06	− 0.35	Toscana	Pisa	0.27	0.42	0.17
Piemonte	Alessandria	− 0.27	0.06	− 0.25	Toscana	Arezzo	0.18	-0.23	− 0.11
Piemonte	Biella	− 0.08	0.26	− 0.27	Toscana	Siena	0.31	0.18	0.07
Piemonte	Verbano C.O.	−0.07	− 0.13	− 0.62	Toscana	Grosseto	−0.05	− 0.32	− 0.73
Valle d’Aosta	Aosta	0.27	− 0.08	− 0.24	Toscana	Prato	0.44	0.13	− 0.14
Liguria	Imperia	−0.02	− 0.49	− 0.83	Umbria	Perugia	0.02	0.03	− 0.23
Liguria	Savona	0.09	0.13	− 0.29	Umbria	Terni	− 0.34	− 0.20	− 0.44
Liguria	Genova	0.12	0.37	− 0.09	Lazio	Viterbo	− 0.60	− 0.29	−0 .75
Liguria	La Spezia	− 0.12	0.04	− 0.27	Lazio	Rieti	− 0.55	− 0.40	− 1.04
Lombardia	Varese	0.37	0.16	0.29	Lazio	Roma	− 0.11	0.72	0.53
Lombardia	Como	0.43	0.01	0.12	Lazio	Latina	− 0.52	− 0.32	− 0.42
Lombardia	Sondrio	0.03	0.09	− 0.47	Lazio	Frosinone	− 0.59	− 0.49	− 0.62
Lombardia	Milano	0.94	1.57	1.97	Campania	Caserta	− 0.94	− 0.87	− 0.91
Lombardia	Bergamo	0.28	0.24	0.09	Campania	Benevento	− 1.09	− 0.56	− 0.64
Lombardia	Brescia	0.39	0.15	0.08	Campania	Napoli	− 0.46	− 0.72	− 0.36
Lombardia	Pavia	0.18	0.37	0.18	Campania	Avellino	−0.58	− 0.37	− 0.72
Lombardia	Cremona	0.19	0.07	0.03	Campania	Salerno	0.93	0.67	0.52
Lombardia	Mantova	0.03	− 0.14	− 0.17	Abruzzo	L’Aquila	− 0.39	0.02	− 0.76
Lombardia	Lecco	0.57	0.19	0.14	Abruzzo	Teramo	− 0.70	− 0.19	− 0.54
Lombardia	Lodi	0.19	− 0.08	− 0.24	Abruzzo	Pescara	− 0.57	− 0.20	− 0.19
Lombardia	Monza Brianza	0.49	0.39	0.38	Abruzzo	Chieti	− 0.47	− 0.21	− 0.47
Trentino Alto Adige	Bolzano	0.27	0.47	0.18	Molise	Campobasso	− 0.41	− 0.54	− 0.83
Trentino Alto Adige	Trento	0.72	0.44	0.37	Molise	Isernia	− 0.48	− 0.17	− 1.00
Veneto	Verona	0.38	0.29	0.22	Puglia	Foggia	− 0.84	− 0.93	− 0.95
Veneto	Vicenza	0.47	0.26	0.57	Puglia	Bari	− 0.14	− 0.37	− 0.23
Veneto	Belluno	0.36	0.16	− 0.11	Puglia	Taranto	− 0.42	− 0.86	− 0.54
Veneto	Treviso	0.61	0.25	0.26	Puglia	Brindisi	− 0.40	− 0.82	− 0.79
Veneto	Venezia	0.71	0.35	− 0.04	Puglia	Lecce	− 0.65	− 0.70	− 0.64
Veneto	Padova	0.48	0.47	0.35	Puglia	Barletta A.T.	− 0.78	− 0.94	− 1.01
Veneto	Rovigo	0.13	0.09	− 0.55	Basilicata	Potenza	− 0.57	− 0.62	− 0.37
Friuli Venezia Giulia	Udine	0.47	0.42	0.10	Basilicata	Matera	− 0.58	− 0.49	− 0.71
Friuli Venezia Giulia	Gorizia	0.16	− 0.26	0.05	Calabria	Cosenza	− 0.87	− 0.84	− 0.76
Friuli Venezia Giulia	Trieste	0.34	0.78	0.49	Calabria	Catanzaro	− 0.88	− 0.43	− 0.64
Friuli Venezia Giulia	Pordenone	0.32	0.10	0.41	Calabria	Reggio Calabria	− 0.68	− 1.04	− 0.89
Emilia Romagna	Piacenza	0.01	0.38	0.03	Calabria	Crotone	− 1.05	− 1.24	− 0.96
Emilia Romagna	Parma	0.06	0.70	0.44	Calabria	Vibo Valentia	− 0.69	− 1.00	− 0.71
Emilia Romagna	Reggio Emilia	0.43	0.13	0.26	Sicilia	Trapani	− 0.54	− 1.09	− 0.93
Emilia Romagna	Modena	0.20	0.48	0.69	Sicilia	Palermo	− 0.40	− 0.83	− 0.55
Emilia Romagna	Bologna	0.59	0.83	0.92	Sicilia	Messina	− 0.67	− 0.91	− 0.72
Emilia Romagna	Ferrara	− 0.11	0.43	− 0.05	Sicilia	Agrigento	− 0.71	− 1.02	− 1.25
Emilia Romagna	Ravenna	0.44	0.07	0.11	Sicilia	Caltanissetta	− 0.73	− 1.51	− 1.25
Emilia Romagna	Forli Cesena	0.38	0.10	− 0.01	Sicilia	Enna	− 0.51	− 1.12	− 1.20
Emilia Romagna	Rimini	0.22	− 0.27	0.31	Sicilia	Catania	− 0.48	− 0.99	− 0.59
Marche	Pesaro Urbino	− 0.12	− 0.02	− 0.01	Sicilia	Ragusa	− 0.45	− 1.11	− 0.66
Marche	Ancona	0.21	0.10	0.39	Sicilia	Siracusa	− 0.72	− 1.17	− 0.53
Marche	Macerata	0.13	− 0.09	− 0.25	Sardegna	Sassari	− 0.32	− 0.58	− 0.76
Marche	Ascoli Piceno	0.06	0.00	− 0.08	Sardegna	Nuoro	− 0.49	− 0.74	− 1.00
Marche	Fermo	− 0.02	− 0.14	− 0.06	Sardegna	Cagliari	0.24	0.06	− 0.30
Toscana	Massa Carrara	− 0.25	− 0.06	− 0.37	Sardegna	Oristano	− 0.21	− 0.41	− 1.04

With respect to the Basic subindex, the first ten provinces are Milano, Trento, Venezia, Treviso, Bologna, Lecco, Firenze, Monza Brianza, Padova, Udine; the last ten are Siracusa, Caltanissetta, Barletta Andria Trani, Foggia, Cosenza, Catanzaro, Salerno, Caserta, Crotone, Benevento.

With respect to the Efficiency subindex, the first ten provinces are Milano, Bologna, Trieste, Roma, Parma, Firenze, Torino, Modena, Bolzano, Padova; the last ten are Catania, Vibo Valentia, Agrigento, Reggio Calabria, Trapani, Ragusa, Enna, Siracusa, Crotone, Caltanissetta.

With respect to the Innovation subindex, the first ten provinces are Milano, Bologna, Torino, Modena, Vicenza, Firenze, Roma, Trieste, Parma, Pordenone; the last ten are Foggia, Crotone, Isernia, Nuoro, Barletta Andria Trani, Rieti, Oristano, Enna, Caltanissetta, Agrigento.

## Fuzzy Clustering with Multivariate Data and *contiguity* Constraints

The data set can be represented as a spatial data matrix (D’Urso 2000, 2004, 2005) as:

1 $$\begin{aligned} {\mathbf {X}}\equiv \{x_{ij}:i=1,\ldots ,I;\;j=1,\ldots ,J\} \end{aligned}$$

where *i* indicates the generic unit (geographical area or region, i.e. the province), *j* the variable (i.e. the pillar); $x_{ij}$ is the value of the *j*-th variable observed for the *i*-th unit, or alternatively as follows:

2 $$\begin{aligned} {\mathbf {x}}_i\equiv \{x_{ij}:\;j=1,\ldots ,J\} . \end{aligned}$$

Furthermore, we also assume to have *K* additional information on spatial location of the units, i.e. *K* different levels of contiguity. In particular, we can consider *K* $(I\times I)$ symmetric data matrices ${\mathbf {P}}_k\;(k=1,\ldots ,K)$, whose generic entry $p_{kii'}$ is a measure of a particular kind of spatial proximity between the *i*-th and $i'$-th units ($i,i'=1,\ldots ,I$) (Pham 2001; Coppi et al. 2010). In the literature, there are different ways of defining neighbourhood and consequently there are different ways of constructing proximity matrices among spatial units (Gordon 1999; Páez and Scott 2005). Two of the most common definitions are based on connectivity, i.e. travel time or distance between pairs of units, and physical contiguity. Contiguity can be specified in several ways. For instance, two spatial units can be contiguous either if they are adjacent (neighbours) or if they belong to the same macro-area, even if they are not adjacent. In this case, ${\mathbf {P}}$ is constructed as a symmetric matrix with 0 diagonal elements and with off-diagonal elements given by:

3 $$\begin{aligned} p_{ii'}= {\left\{ \begin{array}{ll} 1&\quad \text { if } i \text { is contiguous to } i'\\ 0&\quad \text { otherwise} \end{array}\right. } \quad i=1,\ldots ,I,\;i\ne i'. \end{aligned}$$

The clustering procedure adopted enjoys the benefits connected to Fuzzy clustering and to Partitioning Around Medoids (PAM). Due to the difficulty in identifying a clear boundary between provinces even belonging to the same region, fuzzy clustering is more attractive than the hard clustering methods. In addition, the memberships indicate whether there is a second-best cluster almost as good as the best one, a scenario which hard clustering methods cannot uncover (Everitt et al. 2011). For more details, see D’Urso (2014).

Following a Partitioning-Around-Medoids (Pham 2001, Kaufman and Rousseeuw (2005)) approach in a fuzzy framework, the Fuzzy *C*-Medoids (FCMd) (FCMd, Krishnapuram et al. 2001) clustering algorithm is adopted, thanks of its great advantage of obtaining non-fictitious representative spatial units (i.e. the medoids) as final result. This allows for more appealing and easy to interpret results of the final partition (Kaufman and Rousseeuw 2005). From a computational perspective, fuzzy clustering algorithms are generally more efficient (dramatic changes in the value of cluster membership are less likely to occur in estimation procedures) and they are less affected by both local optima and convergence problems (Everitt et al. 2001; Hwang et al. 2007).

Dealing with spatial data, effects between adjacent units have to be taken into account. Since there could be different, say $K\,(K\ge 1)$, definitions of proximity, *K* spatial penalty terms are added to the objective function.

### The Clustering Model

Following Pham (2001); Coppi et al. (2010); D’Urso et al. (2019a), the Fuzzy C-Medoids clustering algorithm with multivariate data and *contiguity* constraints is then formalised as follows:

4 $$\begin{aligned} \begin{aligned} \min :&\sum \limits _{i=1}^{I}\sum \limits _{c=1}^{C}u_{ic}^m d({\mathbf {x}}_i,\widetilde{{\mathbf {x}}}_{c}) +\sum \limits _{k=1}^{K}\frac{\beta _k}{2}\sum \limits _{i=1}^{I}\sum \limits _{c=1}^{C}u_{ic}^m \sum \limits _{i'=1}^{I}\sum \limits _{{c'\in C_c}}p_{kii'}u^m_{i'c'}\\ s.t.&\sum \limits _{c=1}^{C}u_{ic}=1,\;u_{ic}\ge 0 \end{aligned} \end{aligned}$$

where ${\mathbf {x}}_i$ and $\widetilde{{\mathbf {x}}}_c$ represents the multivariate *i*-th spatial unit and *c*-th spatial medoid $(c=1,\ldots ,C)$, respectively; $d(\cdot ,\cdot )$ is the squared euclidean distance; $m>1$ is the fuzziness parameter; $\beta _k\ge 0$ is the tuning parameter of the *k*-th spatial information; $p_{kii'}$ is the generic element of the $(I\times I)$ “proximity” matrix ${\mathbf {P}}_k$; $C_c$ is the set of the *C* clusters, with the exclusion of cluster *c*; $u_{ic}$ is the membership degree of the unit *i* to the cluster *c*.

The optimal iterative solution of the objective function in 4 is:

5 $$\begin{aligned} u_{ic}=\frac{ \left[ d({\mathbf {x}}_i,\widetilde{{\mathbf {x}}}_{c})+ \sum \limits _{k=1}^{K}\beta _k \sum \limits _{i'=1}^{I} \sum \limits _{{c'\in C_c}}p_{kii'}u^m_{i'c'} \right] ^ {-\frac{1}{m-1}} }{\sum \limits _{c'=1}^{C} \left[ d({\mathbf {x}}_i,\widetilde{{\mathbf {x}}}_{c'})+ \sum \limits _{k=1}^{K}\beta _k \sum \limits _{i'=1}^{I} \sum \limits _{{c''\in C_{c'}}}p_{kii'}u^m_{i'c''} \right] ^{-\frac{1}{m-1}} } \ . \end{aligned}$$

The first term in (4) is the within cluster dispersion due to the multivariate features. The second (spatial dependent) term in (4) suitably allows the objective function to incorporate spatial information. The optimization of the objective function in (4) ensures that the cohesion within clusters is maximized and that the spatial autocorrelation existing in the data at hand is properly coped with.

The second (spatial dependent) term in (4) is the sum of $K\,(K\ge 1)$ spatial penalty terms (Pham 2001; Coppi et al. 2010), one for each definition of proximity among areas considered. In this way, the clustering model captures the information connected to the different levels of the proximity or “contiguity” (multilevel proximity or multilevel “contiguity”). For instance, we can consider the simple case in which the units, i.e. provinces, and macroareas, i.e. regions, are considered. In this specific case, two kinds of proximity (“contiguity”) can be defined, proximity (“contiguity”) among provinces (level 1 proximity or level 1 “contiguity”) and proximity among regions (level 2 proximity or level 2 “contiguity”) which the provinces belong to. Therefore, different scenarios can be identified: (1) two provinces ($a_1$ and $a_2$) are close to each other (level 1 proximity or level 1 “contiguity”) and they belong to the same region (level 2 proximity or level 2 “contiguity”); (2) two provinces ($a_1$ and $b_1$) are close to each other (level 1 proximity or level 1 “contiguity”) but they don’t belong to the same region; (3) two provinces ($a_1$ and $a_3$) are not close to each other but they belong to the same region (level 2 proximity or level 2 “contiguity”); (4) two provinces ($a_1$ and $b_2$) are not close to each other and they don’t belong to the same region.

In each spatial penalty term, two parameters are relevant, the proximity matrix ${\mathbf {P}}_k$, and the tuning parameter $\beta _k$. The role of the *k*-th proximity matrix is to increase the membership degree of unit *i* in cluster *c* and, at the same time, to increase the membership degrees of the units that are connected, in some way, to *i* in cluster *c*, while reducing these membership degrees in the other clusters. We define this spatial smoothing as neighbouring effect, where, as previously observed, the concept of neighbour is vast enough to encompass different types of connectivity between areas. The tuning parameter $\beta _k$ can enhance the neighbouring effect due to ${\mathbf {P}}_k$ if the spatial autocorrelation between units is high, i.e., if the features of a spatial unit display a certain degree of concordance with those of the “neighbour”. Otherwise, $\beta _k$ could counterbalance, if not neutralise at all, the neighbouring effect, if there is relatively low spatial autocorrelation between areas. The choice of the value of $\beta _k$ is data dependent. As observed by Coppi et al. (2010), the choice should be made according to a measure of a within cluster spatial autocorrelation (see Sect. 3.3), to avoid that the spatial smoothing induced by the proximity matrix overcome the cluster separation. Indeed, an excessively high value of one or more $\beta _k$’s could constraint all “neighbour” units to be classified in one cluster, regardless the features observed.

An heuristic procedure for a suitable choice of $\beta _k$ is described in Sect. 3.3.

### Validity Measure

In general, internal validity measures provide useful guidelines in the identification of the best partition (as suggested by Handl et al. 2005; D’Urso 2015). A suitable measure for fuzzy clustering algorithm has been proposed by Xie and Beni (1991).

The Xie and Beni cluster validity index (Xie and Beni 1991) is the ratio between compactness and separation among clusters and it can be expressed as:

6 $$\begin{aligned} XB= \frac{\sum \limits _{i=1}^{I}\sum \limits _{c=1}^{C}u_{ic}^{m} d({\mathbf {x}}_i,\widetilde{{\mathbf {x}}}_c)}{I\min \limits _{p\ne q} x(\widetilde{{\mathbf {x}}}_p,\widetilde{{\mathbf {x}}}_q)} \end{aligned}$$

where $(p,q)\in \{1.\ldots ,C\}$. The smaller *XB*, the more compact and separate are the clusters.

### Spatial Autocorrelation

As deeply analized in Coppi et al. (2010), the optimal choice of the value of the parameter $\beta$ is a very complex issue. It has to be set exogenously by means of an heuristic procedure based on the spatial autocorrelation measure introduced in Coppi et al. (2010), that could be seen as a generalization of the Moran’s index. For a chosen value of *C* and *m* and $k=1$, the algorithm is run for increasing values of $\beta$ (chosen in a suitable interval): the optimal $\beta$ value is that maximizes the within cluster spatial autocorrelation. Properly, it maximizes the Global Moran overall spatial autocorrelation measure $\rho _{overall}$ that, for a given partition, is computed as follows:

7 $$\begin{aligned} \rho _{overall}= \frac{\sum _{c=1}^{C} \rho _{c} \, s_{c}}{I} \end{aligned}$$

where $$s_{c}=\sum _{i=1}^{I}u_{ic}$$.

The $\rho _{c}$, the spatial autocorrelation measure for the *c*-th cluster, is computed as:

8 $$\begin{aligned} \rho _{c}=\frac{ tr\left[ {{\mathbf {X}}}^{\prime }{\mathbf {U}}_c^{\frac{1}{2}}{\mathbf {P}}{\mathbf {U}}_c^{\frac{1}{2}}{{\mathbf {X}}}\right] }{ tr\left[ {{\mathbf {X}}}^{\prime }{\mathbf {U}}_c^{\frac{1}{2}} diag({\mathbf {P}}^{\prime }{\mathbf {P}}){\mathbf {U}}_c^{\frac{1}{2}}{{\mathbf {X}}}\right] } \end{aligned}$$

where ${\mathbf {U}}_c$ is the square diagonal matrix (of order *I*) of the membership degrees of cluster *c*, and ${\mathbf {P}}$ is the spatial contiguity matrix. The operator $diag(\cdot )$ creates a diagonal matrix whose elements in the main diagonal are the same as those of the square matrix in the argument. If *P* is a contiguity matrix with 0/1 values, every diagonal element contains the number of neighboring units for the associated spatial unit.

As for Moran’s index, also for $\rho _{overall}$, a value of 1 ($-1$) identifies a perfect positive (negative) autocorrelation, while 0 indicates the absence of autocorrelation. Therefore, to higher values of the $\rho _{overall}$ corresponds a better spatial assignment of the units to the clusters. An heuristic procedure for a suitable choice of $\beta$ consists in running the clustering model for increasing values of $\beta$, and choosing that value $\beta _{opt}$ such that $\rho _{overall}$ is maximal.

Moreover, the Fuzzy Moran’s index, as the Moran’s index, can be interpreted as a measure of spatial spill-over effect (Ma et al. 2015; Yang 2012). In the literature, the spatial spill-over effect is considered as the indirect or unintentional effect that a geographical area exerts on other neighbour areas (Yang and Fik 2014). A positive spill-over effect is obtained when an area benefits of their neighbours influence due to the existence of spatial externalities across area.

## Fuzzy C-Medoids Clustering of the Italian Provinces

**Fig. 1:**
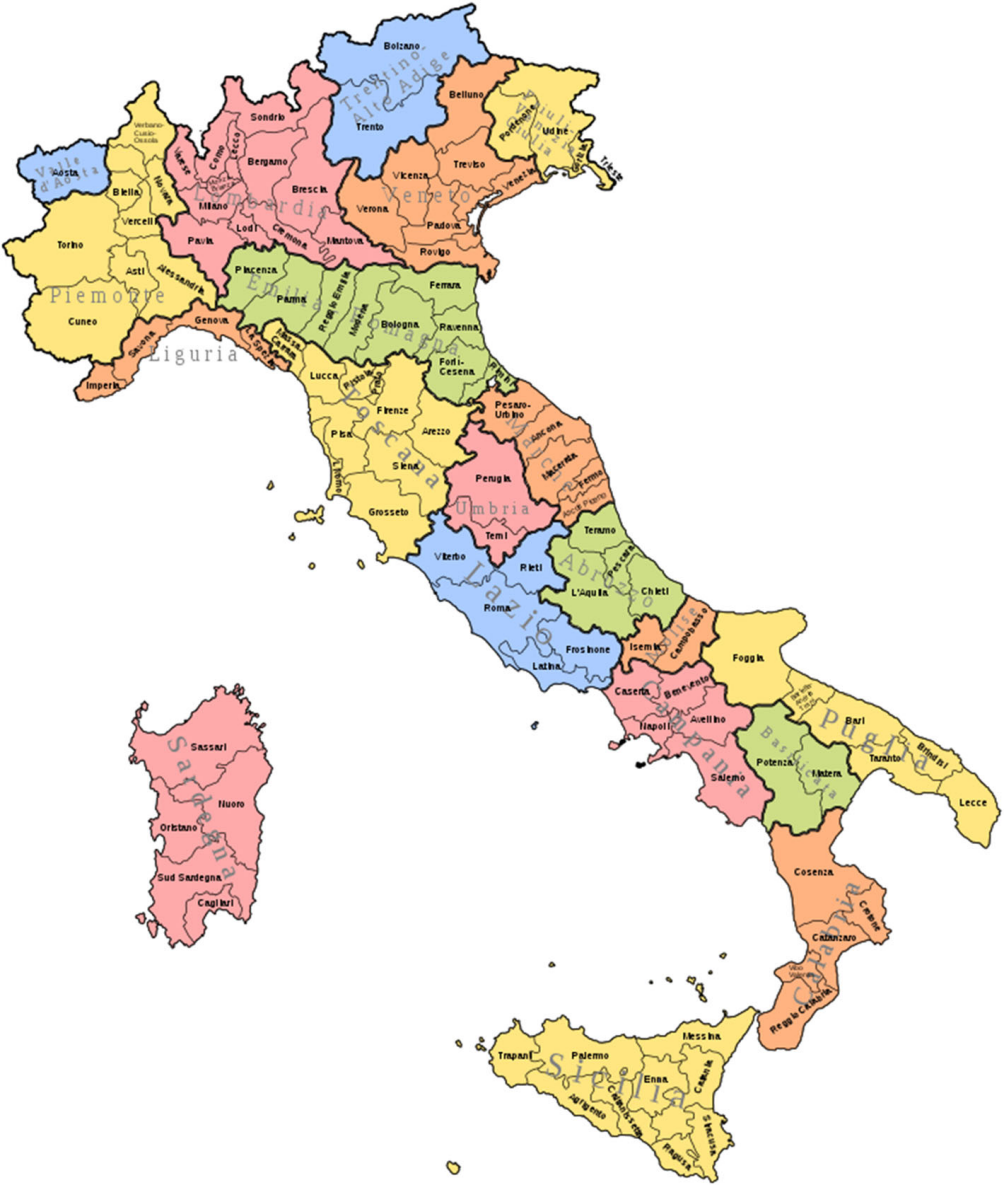
Italian regions and borders of the provinces

The Fuzzy C-Medoid clustering model has been applied to the provinces based on the eleven competitiveness pillars. A number of clusters from 3 to 6 has been considered and the number of clusters has been selected on the basis of the validity criteria illustrated in Sect. 3. The model has been applied without contiguity constraints to set the number of clusters and the value of the fuzziness parameter. On the basis of the value of the Xie-Beni index $C=3$ and $m=1.3$ have been selected. A cut-off of 0.60 for the membership has been considered to determine fuzzy provinces (D’Urso et al. 2015). The original 107 provinces have been reduced to 106 by excluding Sud Sardegna newly established (Fig. 1).

The italian regions are geographically grouped into three areas (Istat):

- North: Liguria, Lombardia, Piemonte, Valle d’Aosta, Emilia-Romagna, Friuli-Venezia Giulia, Trentino-Alto Adige, Veneto;
- Centre: Lazio, Marche, Toscana ed Umbria.
- South and Isles: Abruzzo, Basilicata, Calabria, Campania, Molise, Puglia, Sardegna, Sicilia.

**Fig. 2:**
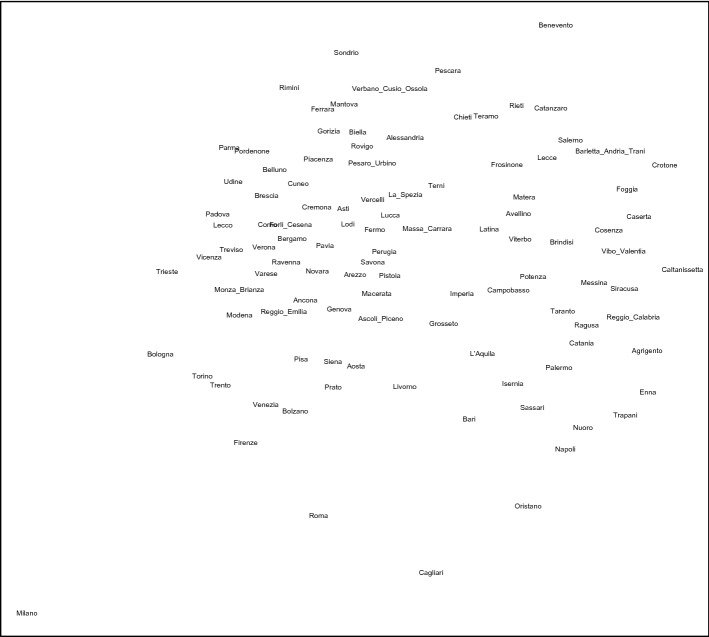
Sammon projection of the provinces on a two-dimensional space

The Sammon projection of the provinces is presented in Fig. 2 (Ghojogh et al. 2020). Three areas are identified. A left area, mostly with the provinces located in the North of Italy; a central area, mostly with the regions in the South-Center of Italy and a right area, mostly with the regions in the South of Italy.

### Fuzzy C-Medoids Clustering of the Italian Provinces

The numerosity of the clusters is: cluster 1 38 provinces, cluster 2 27 provinces, cluster 3 41 provinces.

The medoids are presented in Table 5.

**Table 5:** Fuzzy C-medoids

	Pillar I	Pillar II	Pillar III	Pillar IV	Pillar V	Pillar VI	Pillar VII	Pillar VIII	Pillar IX	Pillar X	Pillar XI
Cluster 1	0.45	0.01	− 0.35	0.18	1.14	0.25	0.37	0.11	− 0.02	0.52	− 0.23
Cluster 2	0.45	− 0.11	− 0.02	− 0.01	0.16	0.78	0.18	− 0.57	− 0.32	− 0.18	− 0.38
Cluster 3	− 0.99	− 0.42	− 0.29	− 0.47	− 0.73	0.05	− 0.62	− 0.54	− 0.80	− 0.50	− 0.87

As a complementary profiling information the average values of the three subindexes within each cluster is computed (Table 6).

**Table 6:** Basic, efficiency and innovation profiling of the clusters

	Basic	Efficiency	Innovation
Cluster 1 (Bergamo)	0.34	0.23	0.21
Cluster 3 (Savona)	0.06	0.12	− 0.15
Cluster 2 (Avellino)	− 0.59	− 0.69	− 0.74

Cluster 1, with medoid Bergamo, is characterised by values of the indicators well over zero. Two Pillars (Pillars II and IX) show values close to zero and one Pillar (Pillar III) just under zero. Provinces in cluster 1 have greatly developed the Basic, Efficiency and Innovation competitiveness subindexes.

Cluster 2, with medoid Savona, is characterised by values of the indicators close to zero o slightly under. Pillars I, V, VI, VII show a positive value. Provinces in cluster 32 have developed the Basic, Efficiency and Innovation competitiveness subindexes at a level in the average of the Italian provinces.

Cluster 3, with medoid Avellino, is characterised by values of the indicators well under zero. One Pillar (Pillar VI) shows a value close to zero. Provinces in cluster 3 show negative values of the Basic, Efficiency and Innovation competitiveness subindexes.

**Fig. 3:**
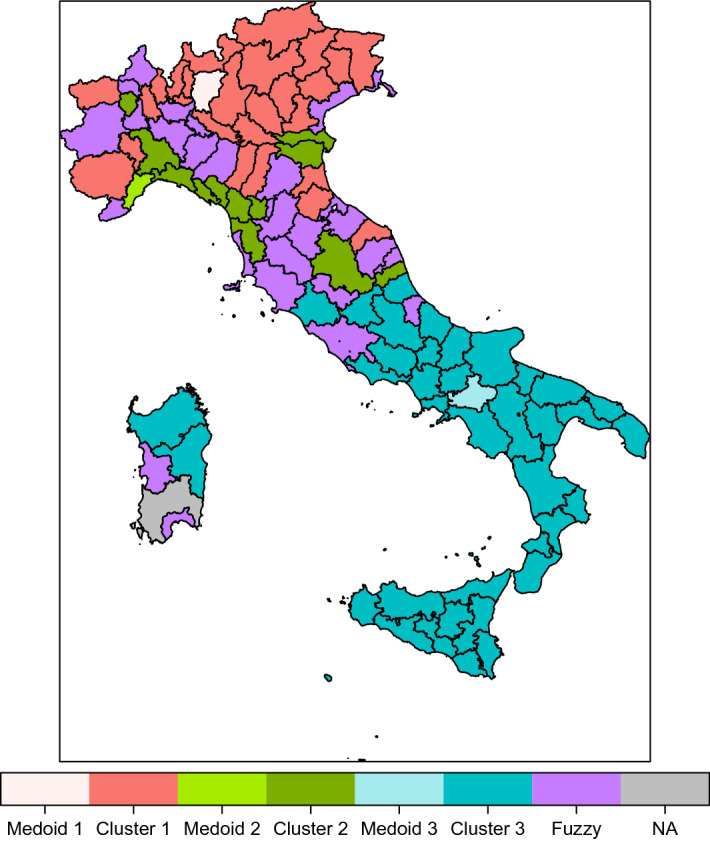
Cartogram cluster representation. Different colors for medoids, clusters and fuzzy provinces

The greatest membership and the related cluster are presented in Table 7 (in bold the medoids) and shown in Fig. 3. Many provinces show a membership under 0.60 (fuzzy provinces). The provinces showing a membership under 0.50 are Imperia, Siena, Roma, Cagliari (in the middle in Fig. 2). Roma and Cagliari, with the lowest memberships, are not in the same cluster of the other provinces of Lazio and Sardegna, respectively, both improving the cluster with respect to the provinces of the same region according to the highest membership.

**Table 7:** Membership and cluster of the provinces

Region	Province	Membership	Cluster	Region	Province	Membership	Cluster
Piemonte	Torino	0.56	1	Toscana	Lucca	0.62	2
Piemonte	Vercelli	0.58	2	Toscana	Pistoia	0.79	2
Piemonte	Novara	0.66	1	Toscana	Firenze	0.52	2
Piemonte	Cuneo	0.87	1	Toscana	Livorno	0.47	2
Piemonte	Asti	0.65	1	Toscana	Pisa	0.68	2
Piemonte	Alessandria	0.65	2	Toscana	Arezzo	0.53	1
Piemonte	Biella	0.60	2	Toscana	Siena	0.48	1
Piemonte	Verbano C.O.	0.49	2	Toscana	Grosseto	0.50	2
Valle d’Aosta	Aosta	0.66	1	Toscana	Prato	0.50	2
Liguria	Imperia	0.45	3	Umbria	Perugia	0.70	2
Liguria	**Savona**	**1.00**	**2**	Umbria	Terni	0.50	2
Liguria	Genova	0.75	2	Lazio	Viterbo	0.79	3
Liguria	La Spezia	0.83	2	Lazio	Rieti	0.85	3
Lombardia	Varese	0.89	1	Lazio	Roma	0.40	1
Lombardia	Como	0.90	1	Lazio	Latina	0.94	3
Lombardia	Sondrio	0.62	1	Lazio	Frosinone	0.94	3
Lombardia	Milano	0.50	1	Campania	Caserta	0.88	3
Lombardia	**Bergamo**	**1.00**	**1**	Campania	Benevento	0.74	3
Lombardia	Brescia	0.93	1	Campania	Napoli	0.66	3
Lombardia	Pavia	0.51	1	Campania	**Avellino**	**1.00**	**3**
Lombardia	Cremona	0.88	1	Campania	Salerno	0.91	3
Lombardia	Mantova	0.63	1	Abruzzo	L’Aquila	0.74	3
Lombardia	Lecco	0.83	1	Abruzzo	Teramo	0.87	3
Lombardia	Lodi	0.57	1	Abruzzo	Pescara	0.52	3
Lombardia	Monza Brianza	0.93	1	Abruzzo	Chieti	0.72	3
Trentino Alto Adige	Bolzano	0.68	1	Molise	Campobasso	0.77	3
Trentino Alto Adige	Trento	0.71	1	Molise	Isernia	0.77	3
Veneto	Verona	0.95	1	Puglia	Foggia	0.90	3
Veneto	Vicenza	0.90	1	Puglia	Bari	0.61	3
Veneto	Belluno	0.91	1	Puglia	Taranto	0.83	3
Veneto	Treviso	0.90	1	Puglia	Brindisi	0.82	3
Veneto	Venezia	0.50	2	Puglia	Lecce	0.85	3
Veneto	Padova	0.87	1	Puglia	Barletta A.T.	0.84	3
Veneto	Rovigo	0.63	2	Basilicata	Potenza	0.81	3
Friuli Venezia Giulia	Udine	0.67	1	Basilicata	Matera	0.88	3
Friuli Venezia Giulia	Gorizia	0.58	1	Calabria	Cosenza	0.86	3
Friuli Venezia Giulia	Trieste	0.52	2	Calabria	Catanzaro	0.85	3
Friuli Venezia Giulia	Pordenone	0.78	1	Calabria	Reggio Calabria	0.80	3
Emilia Romagna	Piacenza	0.58	2	Calabria	Crotone	0.76	3
Emilia Romagna	Parma	0.53	1	Calabria	Vibo Valentia	0.82	3
Emilia Romagna	Reggio Emilia	0.77	1	Sicilia	Trapani	0.70	3
Emilia Romagna	Modena	0.73	1	Sicilia	Palermo	0.77	3
Emilia Romagna	Bologna	0.56	1	Sicilia	Messina	0.88	3
Emilia Romagna	Ferrara	0.81	2	Sicilia	Agrigento	0.80	3
Emilia Romagna	Ravenna	0.86	1	Sicilia	Caltanissetta	0.76	3
Emilia Romagna	Forli Cesena	0.75	1	Sicilia	Enna	0.75	3
Emilia Romagna	Rimini	0.56	1	Sicilia	Catania	0.80	3
Marche	Pesaro Urbino	0.59	2	Sicilia	Ragusa	0.74	3
Marche	Ancona	0.61	1	Sicilia	Siracusa	0.79	3
Marche	Macerata	0.54	2	Sardegna	Sassari	0.69	3
Marche	Ascoli Piceno	0.79	2	Sardegna	Nuoro	0.69	3
Marche	Fermo	0.59	2	Sardegna	Cagliari	0.39	2
Toscana	Massa Carrara	0.66	2	Sardegna	Oristano	0.60	3

Roma shows values of the subindexes Basic, Efficiency, Education well over the values of the provinces in the same cluster (Table 4). The strengths, considering the pillars, are: in the Basic subindex Infrastructure; in the Efficiency subindex Higher Education, Labor market Efficiency and Market Size; in the subindex Innovation, Technological Readiness and Innovation (due to public financial support to Research and Development). Explanations of the low membership to cluster 1 are the following. The weakness in the other pillar of the subindex Innovation is due to the fact that the business sector is less important than in most of the other central and northern Italian provinces and is very much oriented towards non market services (Public Administration at national level). About 84% of its value added (at current market prices) is related to services, the highest share among the Italian provinces, of which 39% to financial and insurance, real estate, professional, scientific and technical activities. The weakness in the other pillars of the subindex Basic is due to shortcomings in the economic fundamentals (Table 13).

Cagliari shows values of the subindexes Basic, Efficiency, Innovation well over the values of the provinces in the same cluster. The strengths are: in the Basic subindex Macroeconomic stability and Health; in the Efficiency subindex Higher Education. The local economic system is characterized by strong economic fundamentals, above all the solidity in the local finance. The main weakness is the small internal demand and the presence of micro enterprises. The creation of the Digital Innovation Hub (DIH) has the mission of enhancing and networking the various actors of the digital Innovation ecosystem to strengthen the manufacturing vocation of the territory and by doing so, make Industry 4.0 the driving force for development and competitiveness for the local and regional economy. Explanations of the low membership to cluster 2 are the shortcomings in Basic Education in the Basic subindex and of Technological Readiness and Innovation in the Innovation subindex.

Milano shows a membership 0.50 (at the lower left edge in Fig. 2). The reason of the low membership to cluster 1 is due to the highest scores in all the pillars of the subindexes Basic, Efficiency, Innovation with respect to the other provinces. Milano, in addition to presenting strong fundamentals and high indicators of efficiency of the production system, has a knowledge-based economy with a high propensity for research and development and a high ability to retain talent and attract talent from other territories.

The regions Emilia Romagna, Friuli Venezia Giulia, Lazio, Liguria, Marche, Piemonte, Sardegna, Toscana, Veneto show provinces in different clusters. Some comments on the position of Ancona, not in the same cluster of the other (even contiguous) provinces of the region Marche. Ancona shows values of the subindexes Basic, Efficiency, Education well over the values of the the provinces in the same cluster. It shows high membership to cluster 1. The strengths are: in the Basic subindex Institutions, Health and Basic Education; in the Efficiency subindex Higher Education and Labor market Efficiency; in the subindex Innovation Technological Readiness and Business Sophistication. At present there is no advanced, knowledge intensive service sector which is instrumental in increasing the propensity to invest in research and technology, that limits growth in the Innovation pillar.

The analysis could be also deepened considering the elemenatry indicators within each pillar.

The contribution of the regions to the clusters is presented in Table 8. Ten regions contribute to cluster 1, all located in the North area of Italy except Lazio (Roma province) and Marche (Ancona province). Nine regions contribute to cluster 2, located in the North, Centre and South areas. Ten regions contribute to cluster 3, all located in the South area except Lazio and Abruzzo. All the provinces of the regions Lombardia, Trentino Alto Adige, Valle d’Aosta are assigned to cluster 1.

**Table 8:** Region contribution to clusters

Region	Cluster 1	Cluster 2	Cluster 3	Total
Abruzzo			4	4
Basilicata			2	2
Calabria			5	5
Campania			5	5
Emilia Romagna	7	2		9
Friuli Venezia Giulia	3	1		4
Lazio	1		4	5
Liguria		3	1	4
Lombardia	12			12
Marche	1	4		5
Molise			2	2
Piemonte	4	4		8
Puglia			6	6
Sardegna		1	3	4
Sicilia			9	9
Toscana	2	8		10
Trentino Alto Adige	2			2
Umbria		2		2
Valle d’Aosta	1			1
Veneto	5	2		7
total	38	27	41	106

**Fig. 4:**
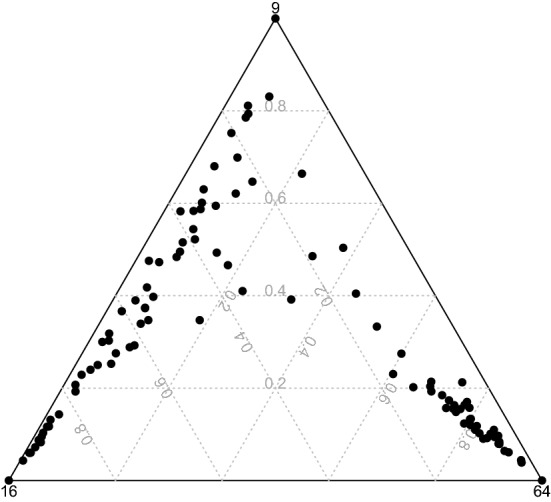
Ternary plot

The ternary plot of the memberships is presented in Fig. 4. It shows fuzzy provinces.

### Fuzzy C-Medoids Clustering of the Italian Provinces with Contiguity Constraints

A contiguity matrix describing the presence of geographic contiguity among provinces has been introduced in the model, taking into account only one level of contiguity ($k=1$). The model with $C=3$ and $m=1.3$ has been applied for a vector $\beta$ of values from 0 to 2 step 0.1, and the value of $\beta$ corresponding to the greatest $\rho _{overall}$ index has been selected. A value of $\beta =0.8$ has been chosen, related to a correlation value $\rho _{overall}=0.53$.

The numerosity of the clusters is: cluster 1 55 provinces, cluster 2 10 provinces, cluster 3 41 provinces.

The medoids are presented in Table 9.

**Table 9:** Fuzzy C-medoids with contiguity constraints

	Pillar I	Pillar II	Pillar III	Pillar IV	Pillar V	Pillar VI	Pillar VII	Pillar VIII	Pillar IX	Pillar X	Pillar XI
Cluster 1	0.45	0.01	− 0.35	0.18	1.14	0.25	0.37	0.11	− 0.02	0.52	− 0.23
Cluster 2	0.03	− 0.25	− 0.50	0.20	0.40	0.32	0.00	− 0.74	− 0.30	0.53	− 0.42
Cluster 3	− 0.99	− 0.42	− 0.29	− 0.47	− 0.73	0.05	− 0.62	− 0.54	− 0.80	− 0.50	− 0.87

As a complementary profiling information the average values of the three subindexes within each cluster is computed (Table 10).

**Table 10:** Basic, Efficiency and Innovation profiling of the clusters - contiguity

	Basic	Efficiency	Innovation
Cluster 1 (Bergamo)	0.25	0.15	0.10
Cluster 3 (Fermo)	0.06	− 0.10	− 0.18
Cluster 2 (Avellino)	− 0.59	− 0.60	− 0.72

Overwhelmingly, with respect to the partitioning without spatial contraints in which there is one cluster with very good, one with medium and one with low competitiveness, the grouping of provinces in the same geographic area gives rise to one cluster with very good and two with low/very low competitiveness.

Cluster 1, has medoid Bergamo, as in the partition without spatial constraint. The average value of the Innovation subindex is smaller than in cluster 1 without contiguity constraints, being the medoid the same. We underline that with respect to the partition without contiguity constraints Roma, which has among the greatest values of the indicators in the subindexes Efficiency and Innovation, has moved to cluster 3.

Cluster 2, with medoid Fermo, is characterised by values of the indicators under zero. Pillars IV, V, VI, X show a positive values. Provinces in cluster 2 show negative values of the Efficiency and Innovation competitiveness subindexes.

Cluster 3, has medoid Avellino, as in the partition without spatial constraint.

**Fig. 5:**
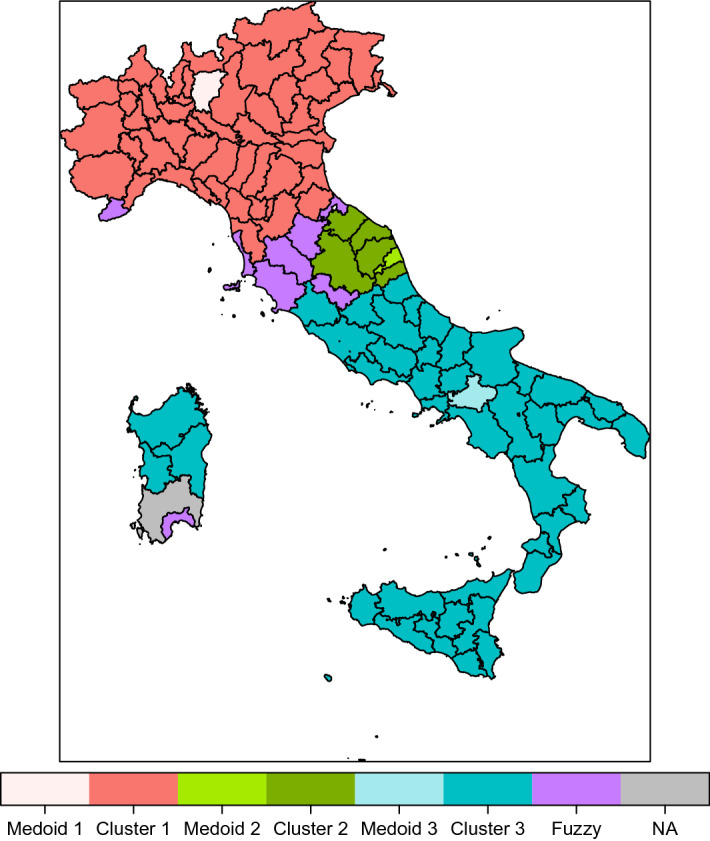
Cartogram cluster representation - contiguity constraint. Different colors for medoids, clusters and fuzzy provinces. (Color figure online)

The greatest membership and the cluster are presented in Table 11 (in bold the medoids) and shown in Fig. 5. There is only one province, Cagliari, showing membership under 0.50. Overall, the contiguity constraint forces the contiguous provinces, generally located in the same region, in the same cluster. Few provinces violate the contiguity within the region: Arezzo with respect to contiguos provinces in Toscana; Rimini with respect to contiguos provinces in Emilia Romagna.

**Table 11:** Membership and cluster of the provinces - contiguity

Region	Province	Membership	Cluster	Region	Province	Membership	Cluster
Piemonte	Torino	0.97	1	Toscana	Lucca	0.96	1
Piemonte	Vercelli	0.99	1	Toscana	Pistoia	0.96	1
Piemonte	Novara	0.99	1	Toscana	Firenze	0.94	1
Piemonte	Cuneo	0.98	1	Toscana	Livorno	0.50	1
Piemonte	Asti	0.99	1	Toscana	Pisa	0.79	1
Piemonte	Alessandria	0.97	1	Toscana	Arezzo	0.54	2
Piemonte	Biella	0.92	1	Toscana	Siena	0.52	1
Piemonte	Verbano C.O.	0.89	1	Toscana	Grosseto	0.38	1
Valle d’Aosta	Aosta	0.95	1	Toscana	Prato	0.90	1
Liguria	Imperia	0.58	1	Umbria	Perugia	0.91	2
Liguria	Savona	0.94	1	Umbria	Terni	0.46	2
Liguria	Genova	0.97	1	Lazio	Viterbo	0.79	3
Liguria	La Spezia	0.86	1	Lazio	Rieti	0.83	3
Lombardia	Varese	0.99	1	Lazio	Roma	0.80	3
Lombardia	Como	0.99	1	Lazio	Latina	0.99	3
Lombardia	Sondrio	0.98	1	Lazio	Frosinone	1.00	3
Lombardia	Milano	0.88	1	Campania	Caserta	0.99	3
Lombardia	**Bergamo**	**1.00**	**1**	Campania	Benevento	0.97	3
Lombardia	Brescia	1.00	1	Campania	Napoli	0.94	3
Lombardia	Pavia	0.99	1	Campania	**Avellino**	**1.00**	**3**
Lombardia	Cremona	1.00	1	Campania	Salerno	0.98	3
Lombardia	Mantova	0.99	1	Abruzzo	L’Aquila	0.99	3
Lombardia	Lecco	0.99	1	Abruzzo	Teramo	0.92	3
Lombardia	Lodi	0.98	1	Abruzzo	Pescara	0.90	3
Lombardia	Monza Brianza	1.00	1	Abruzzo	Chieti	0.97	3
Trentino Alto Adige	Bolzano	0.94	1	Molise	Campobasso	0.99	3
Trentino Alto Adige	Trento	0.98	1	Molise	Isernia	0.99	3
Veneto	Verona	1.00	1	Puglia	Foggia	0.99	3
Veneto	Vicenza	0.99	1	Puglia	Bari	0.96	3
Veneto	Belluno	1.00	1	Puglia	Taranto	0.97	3
Veneto	Treviso	1.00	1	Puglia	Brindisi	0.96	3
Veneto	Venezia	0.96	1	Puglia	Lecce	0.95	3
Veneto	Padova	0.99	1	Puglia	Barletta A.T.	0.96	3
Veneto	Rovigo	0.98	1	Basilicata	Potenza	0.99	3
Friuli Venezia Giulia	Udine	0.98	1	Basilicata	Matera	0.99	3
Friuli Venezia Giulia	Gorizia	0.82	1	Calabria	Cosenza	0.98	3
Friuli Venezia Giulia	Trieste	0.69	1	Calabria	Catanzaro	0.98	3
Friuli Venezia Giulia	Pordenone	0.98	1	Calabria	Reggio Calabria	0.92	3
Emilia Romagna	Piacenza	0.99	1	Calabria	Crotone	0.89	3
Emilia Romagna	Parma	0.98	1	Calabria	Vibo Valentia	0.93	3
Emilia Romagna	Reggio Emilia	0.99	1	Sicilia	Trapani	0.87	3
Emilia Romagna	Modena	0.99	1	Sicilia	Palermo	0.97	3
Emilia Romagna	Bologna	0.96	1	Sicilia	Messina	0.97	3
Emilia Romagna	Ferrara	0.95	1	Sicilia	Agrigento	0.94	3
Emilia Romagna	Ravenna	0.99	1	Sicilia	Caltanissetta	0.96	3
Emilia Romagna	Forli Cesena	0.88	1	Sicilia	Enna	0.95	3
Emilia Romagna	Rimini	0.49	2	Sicilia	Catania	0.97	3
Marche	Pesaro Urbino	0.93	2	Sicilia	Ragusa	0.93	3
Marche	Ancona	0.94	2	Sicilia	Siracusa	0.91	3
Marche	Macerata	0.99	2	Sardegna	Sassari	0.85	3
Marche	Ascoli Piceno	0.86	2	Sardegna	Nuoro	0.84	3
Marche	**Fermo**	**1.00**	**2**	Sardegna	Cagliari	0.37	2
Toscana	Massa Carrara	0.87	1	Sardegna	Oristano	0.80	3

The contribution of the regions to the clusters is presented in Table 12. Nine regions contribute to cluster 1, all located in the North area of Italy except Toscana. Five regions contribute to cluster 2, all located in the Centre and South areas. Nine regions contribute to cluster 3, all located in the South area. As a general comment provinces in the same region are assigned to the same cluster.

**Table 12:** Region contribution to clusters

Region	Cluster 1	Cluster 2	Cluster 3	Total
Abruzzo			4	4
Basilicata			2	2
Calabria			5	5
Campania			5	5
Emilia Romagna	8	1		9
Friuli Venezia Giulia	4			4
Lazio			5	5
Liguria	4			4
Lombardia	12			12
Marche		5		5
Molise			2	2
Piemonte	8			8
Puglia			6	6
Sardegna		1	3	4
Sicilia			9	9
Toscana	9	1		10
Trentino Alto Adige	2			2
Umbria		2		2
Valle d’Aosta	1			1
Veneto	7			7
total	55	10	41	106

**Fig. 6:**
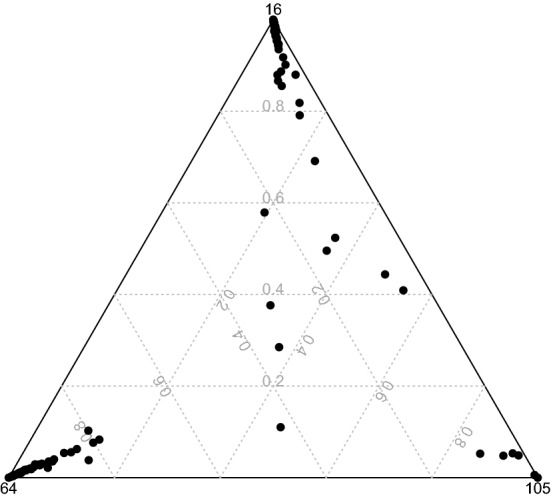
Ternary plot

The ternary plot of the memberships is presented in Fig. 6. It shows very few fuzzy provinces.

## Conclusions

In this paper indicators of attractiveness at NUTS3 level in the framework of the EU Regional Competitiveness Index (RCI) are proposed. Then the Fuzzy C-Medoids Clustering model with multivariate data and *contiguity* constraints is applied for partitioning the Italian provinces (NUTS3). The novelty is the territorial level analized, and the identification of the indicators at the basis of the construction of the eleven composite competitiveness pillars. A *contiguity* constraint, based on the geographic contiguity of provinces, is also introduced in the model. With respect to the partitioning without spatial contraints in which there is one cluster with very good, one with medium and one with low competitiveness, the grouping of provinces in the same geographic area gives rise to one cluster with very good and two with low/very low competitiveness.

The first contribution of the paper is the territoral dimension of attractiveness. at NUTS3 level. The obtained provincial partitions based on the eleven dimensions - pillars - of attractiveness are not the end point of a statistical exercise in itself, but rather a starting point for an exhaustive reading of our territories. Each composite pillar enables to carry out a precise anamnesis of the territory through the “components” of the pillar, and then to define the “cure” with the formulation of policy proposals tailored to each territory. The added value of the measurement approach adopted lies in its biunivocity: it is possible to move from indicators to pillars and vice versa. In this rewind activity, it is possible to identify the elementary indicator(s) whose value has been decisive in generating a given performance in a particular pillar, that is in a dimension of attractivity.

The second contribution of the paper is the relevance of policies based on contiguity of territories. The analysis has shown that contiguous provinces may be assigned to different clusters, even in the presence of contiguity constraints in the clustering model, showing the relevance of policies based on a NUTS3 level, a route already considered by the Italian government.

The analysis developed and the related set of indicators at NUTS3 level constitute an information base that could be effectively used for the implementation of the National Recovery and Resilience Plan (NRRP). The proposed indicators enrich the information framework at disposal of the policy makers constituted by the BES of the territories (BES-Istat) and can guide the allocation of European resources according to the extent of the territorial gap.

## Appendix

*Pillar I - Institutions* The indicators selected for the analysis are:

- pending trials (reversed). Pending trials of more than three years - civil justice - percentage values of total proceedings. The incidence of proceedings that have not been resolved within the time limits provided by law and that have been in “storage” for more than three years measure the degree of inefficiency of the judicial system, which has a strong impact on the operating costs of the public apparatus. In fact, the parties involved could claim compensation from the State for unreasonable duration and this waste of resources, taken away from strategic investments for the area, explains the negative sign with respect to attractiveness.
- trial duration (reversed). Effective average duration in days of civil proceedings - civil justice. The duration of civil proceedings has historically been a major obstacle to the attraction of foreign direct investment (FDI) of the greenfield type where the company builds new facilities (“green”) such as sales office, production plant that have a strong impact on the territory because of the creation of new jobs, in particular for the cumbersome nature of the resolution of labor disputes between the employer and the employee.
- vote participation. European elections, as a percentage of total eligible voters, average, 2004, 2009, 2014, 2019.
- female municipal administrators out of total local administrators. Gender equity in terms of “representation” is a proxy for the status and role of women in society.
- social relations intensity. Non profit organizations, per 10000 inhabitants. The solidarity networks of associationism are a strength of a territory that makes up for the shortcomings of public services provided at the local level.
- administrative capacity (NUTS3 level). Rating classes: “excellent” score 90-100; “very good” 80-89; “good” 60-79; “satisfactory” 50-59; “weak” 40-49; “poor” 20-39; “fallible” 0–19, (Fondazione Etica on data from Amministrazione Trasparente). The Public Rating evaluates not the policies but the administrative machine that those policies produce. It analyzes, from an ESG (Enviromental, Social, Governance) sustainability perspective, six areas related to the administrative capacity of Public Administrations: Budget, Governance, Personnel Management, Services and relationship with citizens, Procurement and relationship with suppliers, Environment^3^
- corruption in the last 3 years (reversed). Proportion of persons who had at least one contact with a public official and who paid a bribe to a public official, or were asked for a bribe by those public officials, during the last three years before the survey.
- bribe health (reversed). Percentage of persons who know someone (friends, relatives, colleagues) who has been asked for money, favors, gifts in exchange for goods or services in the Health/Care sector when applying for welfare benefits, such as grants, subsidies, social or public housing, disability pensions, or other benefits.
- bribe assistance (reversed). Percentage of people who know someone (friends, relatives, colleagues) who has been asked for money, favors, gifts in exchange for goods or services in the care sector when applying for welfare benefits, such as grants, subsidies, social or public housing, disability pensions, or other benefits.
- bribe education (reversed). Percentage of people who know someone (friends, relatives, colleagues) who have been asked for money, favors, or gifts in exchange for goods or services in the Education sector.
- bribe job (reversed). Percentage of people who know someone (friends, relatives, colleagues) who have been asked for money, favors, or gifts in exchange for goods or services in employment when looking for a job, participating in a contest, or starting a job.
- bribe bribe administration (reversed). Percentage of people who know someone (friends, relatives, colleagues) who have been asked for money, favors, gifts in exchange for goods or services by a public official.^4^

*Pillar II - Macroeconomic stability*

The indicators selected for the analysis are:

- surplus (deficit) of administration in relation to current revenues. With local government revenues steadily exceeding expenditures, a virtuous cycle is triggered whereby a virtuous local PA with a financial margin is able to intervene in the local economy by injecting new resources that raise the attractiveness potential through a positive migration balance and a net flow of new productive settlements.
- collection capacity. Percentage ratio of accrued collections to total collected amount. It measures the ability of the local government to collect assessed revenues and is calculated through the percentage ratio of accrued collections to total collections.
- interest expenses in relation to current revenues (reversed). These are current expenses linked to debts previously contracted by local government, which explains the negative sign with respect to attractiveness.

*Pillar III - Infrastructure*

The indicators selected for the analysis are:

- accessibility (travel times) index towards urban and logistic nodes (reversed). The index is based on matrices of distances in km and average travel times, calculated with GIS instruments. This is because it is not enough to know the actual level of physical infrastructure of a given area, but it is also important to have information regarding its degree of use, its accessibility, its usefulness and the service actually provided.
- seats km offered by all modes of transport per inhabitant. The availability of a local public transport offer (Tpl) adequate to the needs of the population is an essential aspect for the quality of life in cities. A widespread and deficient service reduces traffic congestion and consequently travel time, contains the economic costs incurred by families and businesses and ensures better air quality by reducing the use of private vehicles.
- annual passenger density in local public transport and airports per inhabitant. Ratio of number of passengers in local public transport and airports to population.
- car-sharing: availability of vehicles per 100 thousand inhabitants. Considering that more than 30 million residents move every day in Italy to reach their place of study or work, new forms of travel are developing that involve sharing a car with study or work colleagues.

*Pillar IV - Health*

The indicators selected for the analysis are:

- life expectancy at birth, average number of years. The increase in life expectancy of the population, together with the decrease in the birth rate have strongly characterized Italy in recent decades, with a significant impact on the age structure of the population. Estimates made by Istat (2021) on life expectancy for 2020 indicate that ”following the COVID-19 pandemic that has significantly affected Italy, characterized by a demographic structure much older than other countries, a significant reversal in the process of steady improvement in longevity observed in recent years, especially in some areas of the country particularly affected by the spread of the virus. In terms of life expectancy at birth, compared with an estimate of about 0.9 years lost overall at the national level (from 83.2 to 82.3 years), a strong heterogeneity emerges among the various territories, with an emptying, in terms of years lived, more marked in the northern regions (from 83.6 to 82.1 years expected), compared with the center (from 83.6 to 83.1) and the south (from 82.5 to 82.2).
- infant mortality per 1000 live births (reversed). Ratio of the number of deaths in the first year of life per 10000 live births. The first year of life accounts for 85% of deaths under 5 years of age. Today the mortality rate of children under 5 in Italy is lower than the European average and lower than that of the United States: it has gone from 347 deaths per thousand live births in 1887 to about 4 per thousand today.
- cancer mortality (20–64 years). Standardized rates per 10000 residents (reversed).
- hospital outmigration to other regions for ordinary acute hospitalizations. Proportion of total hospitalized persons residing in the region (reversed). Mobility for health reasons is inversely related to the quality of services offered by the territory of residence, which explains the negative sign of the link with attractiveness.

*Pillar V - Basic Education*

The indicators selected for the analysis are:

- vocational (vocational) graduates. Technical and vocational graduates (proportion of total graduates in the province). Vocational education programmes are designed to provide learners with the knowledge and the set of skills specific to a particular occupation or trade. Such programmes may have work-based components (e.g. apprenticeships, dual-system education programmes). Successful completion of such programmes leads to labour market-relevant, vocational qualifications acknowledged as occupationally oriented by the relevant national authorities and/or the labour market (Eurostat 2020)
- students’ reading proficiency level - mean score. Reading proficiency is essential for a wide variety of human activities—from following instructions in a manual; to figuring out the who, what, when, where and why of a situation; to the many ways of communicating with others for a specific purpose or transaction (OECD - PISA 2018). Proficiency in literacy is closely related to proficiency in numeracy.
- students’ numeracy proficiency level—mean score. Mathematical performance measures the mathematical literacy of a 15 year-old to formulate, employ and interpret mathematics in a variety of contexts to describe, predict and explain phenomena, recognising the role that mathematics plays in the world. The mean score is the measure. A mathematically literate student recognises the role that mathematics plays in the world in order to make well-founded judgments and decisions needed by constructive, engaged and reflective citizens.
- underachievement rate in reading. Percentage of students in grades II of the upper secondary school who do not achieve Level II on (5 levels) in literacy (BES Istat) (reversed). Level 2 is considered the baseline of proficiency, thus the pupils performing under this baseline are considered underachievers (the OECD refers to them as low achievers). This is not only a worrying social issue, but also a drag on Italy future economic competitiveness. Education systems can pursue excellence and equity at the same time: provinces with small proportions of underachievers tend to have also high proportions of top performers.
- underachievement rate in numeracy. Percentage of students in grades II of the upper secondary school who do not achieve Level II on (5 levels) in numeracy (BES Istat) (reversed). Inadequate mathematical proficiency concerns a larger collective of boys (37.8% on average in Italy) than alphabetical proficiency with gender differences: in mathematics, girls did not reach sufficient levels in 42.2% of cases while boys did in 33.5%.

*pillar VI - higher education*

The indicators selected for the analysis are:

- percentage incidence of tertiary graduates 25-39 on the total population in the same cohort. The entire educational pathway is fundamental, but the initial levels of education (elementary and middle school license) are important for the imitation of technologies produced by other economic systems and also for the diffusion of existing technologies to the entire economic system, while the higher levels (upper secondary school diploma and, even more so, university degree) are necessary to generate Innovation and develop new processes and/or products.
- transition to tertiary education. Percentage of recent high school graduates enrolling in university for the first time in the same year they graduated from high school. The continuation of the educational pathway, offers the student the opportunity to enroll in university or other tertiary paths. Only half of new graduates enroll for the first time at university in the same year they graduated from high school. Enrollment, like the outcome of university studies, is strongly influenced by the ease of access to courses (low costs, scholarships), the flexibility of programs, the variety of paths offered and their territorial distribution.
- life long learning. Percentage of people aged 25-64 who participated in education and training in the 4 weeks prior to the interview out of the total number of people aged 25-64. The Skills Agenda indicates that all citizens must have access to attractive, innovative, and inclusive learning programs, even as skills become obsolete very quickly. Learning throughout life, even in old age, will make the difference. In 2020, the opportunity to participate in learning activities was, like school, abruptly interrupted, especially in March, April and May, or partially converted to other forms of educational offerings .
- early school leavers. According to Eurostat, people 18-24 years old who has completed at most lower secondary education and is not involved in further education or training as a percentage of the people aged 18 to 24 out of the total population aged 18 to 24.
- stem graduates. Percentage over total province graduates. The Skills Agenda for Europe proposes in Action 7 to ”increase the number of graduates in STEM disciplines (Science, Technology, Engineering and Mathematics) and promote entrepreneurial and soft skills.

*Pillar VII - Labor market Efficiency*

The indicators selected for the analysis are:

- employment rate 15–64 years. Percentual values of population 15–64 years. The higher the educational attainment level, the higher the employment rate: the level of educational attainment can affect employment rates considerably The employment rate of people (aged 20–64) who had completed a high level of education short-cycle tertiary, bachelor’s, master’s or doctoral levels (or equivalents) (ISCED levels 5–8) was 78.0% was much higher than the rate for those who have only attained education at a low level (primary or lower secondary education, ISCED levels 0–2) which was 50.9% for the EU. The Italian employment rate of people who have completed their education at a medium level, i.e. an upper secondary or post-secondary non-tertiary education (ISCED levels 3–4), is situated between the two previous rates, at 65.1%.
- gender gap - employment rate. Absolute difference between male and female employment rates at ages 15 and over. The difference between the employment rates of men and women of working age (15 and over). Across the EU-27, the gender employment gap was positive, meaning that the proportion of men of working age in employment exceeded that of women. The gender employment gap varies significantly across provinces: the lowest gap was reported in Trieste (4.6 p.p.), followed by Cagliari (6.4 p.p.); the highest in Barletta-Andria-Trani (30.1 p.p.) followed by Trapani (29.1 p.p.). One of the Europe 2020 strategy targets was to have an employment rate for women and men of at least 75% for persons aged 20 to 64 years.
- missing work participation rate (reversed). Percentage ratio of job seekers plus inactive persons immediately available for work (part of the potential labor force) to the corresponding labor force plus inactive persons immediately available for work. Compared to the better known unemployment rate, the non-participation rate provides a broader measure of the labor supply: the numerator includes not only the unemployed but also those who are not looking for work but would be available for work, and the denominator together with the latter also includes the labor force (employed and unemployed). It takes into account those who are available for work even though they are not actively seeking work.
- gender gap - missing work participation. Absolute difference between male and female non participation rate 15-29 years old (reversed). The southern provinces are the most penalized and the highest value is recorded in Sicily, where the rate of non-participation in work is more than double the national value (40.7%).
- share 15—24 not in education, employment, training (neet) (reversed). The share of young people neither in employment nor in education and training is an indicator that measures the proportion of a given subpopulation who are not employed and not involved in any further education or training. These people may be subdivided into those who are unemployed and those who are considered economically inactive (they do not have a job and they are not actively seeking employment).
- labor productivity per person employed. Value added at factor costs divided by the number of persons employed, in euros. The industrial activities (manufacturing, plus electricity, gas, steam andair conditioning supply; water supply, plus waste and remediation and mining and quarrying) contributes more in terms of value added than employment to the overall non-financial business economy, indicating an above average apparent labour productivity.
- formal job. Ratio between the average number of days actually paid in the year to an Inps-insured employee and the theoretical number of days paid in a year to a full-time employee (312 days). The existence of the underground economy in the Italian territories can be caught by means of this ratio. The higher the percentage of the underground economy, the lower the attractiveness.
- fatal accidents at work (reversed). Fatal accidents at work are those that lead to the death of the victim within one year of the accident taking place. Non-fatal accidents at work are defined as those that imply at least four full calendar days of absence from work (they are sometimes also called ‘serious accidents at work’). It is one of the indicators that contribute to measure the quality of work is safety in addition to stability, pay, competence.
- wages of tertiary graduates and doctoral or equivalent. Earnings tend to rise in line with people’s level of education. People with higher (tertiary) education can expect to earn 55.0% more on average than a person without tertiary education. Its variability across provinces could represent a comparative advantage for a territory to get the best talents.

*Pillar VIII - Market size*

The indicators selected for the analysis are:

- provincial gdp year 2017. Chain linked volume, base year 2015. One of the best ways to boost future economic growth is to identify potential markets by expanding the production of goods and services and the turnover of the enterprises
- population (2020) The population size is a proxy of the total customers of a given territory. Age/generation, gender, income, education, ethnicity are the main characteristics that define target customers that make up its share of the total available market. Labor market participation especially among women represents an important factor in the growth of the middle class and also increases the probability of belonging to the middle class which has the highest marginal propensity to consume (MPC).^5^) among the whole population.
- distance of 2017 gdp from pre-crisis gdp levels. Index numbers 2007=100. It is a measure of the resilience of a given territory.
- potential market in terms of share of the province’s gdp in the total italian gdp
- propensity to export. Provincial exports/Provincial GDP*100. Firm characteristics, namely, firm size, openness and the educational level of managers are the key determinants of export propensity in given territory.
- propensity to import. Provincial imports/Provincial GDP*100. A rising income for businesses and households spurs greater demand for goods from abroad and viceversa.
- non-performing loans. Percentage ratio between the amount of the new non-performing loans during the year and the stock of loans not non-performing) (reversed). A nonperforming loan (NPL) is a loan in which the borrower hasn’t made any scheduled payments. An high value of this indicator is signal of weakness in the spending power of the consumers and fragility of the local financial system.

*Pillar IX - Technological readiness*

The indicators selected for the analysis are:

- ultrabroadband penetration. Number of ultrabroadband subscriptions as a percentage of resident population. While ”traditional” broadband over copper or cable TV lines continues its expansion in many provinces, telecommunications operators have started deploying the so-called ”next generation networks” (NGNs), that is, fiber-optic access networks, to provide high-speed broadband services to consumers.
- number of firms registered in the innovative sme section by province per 1000 registered firms. Innovative SMEs are the second evolutionary stage of innovative startups, the so-called ”mature” and ready for the consolidated growth phase.
- manufacturing specialization in high-tech sectors Percentual values of total employees in local units. The term “specialization” refers to the different weight the productive activities have in the production structure of a province. Based on NACE Rev.2. high-technology manufacturing includes the following 3-digit level sectors: $\bullet$ Manufacture of basic pharmaceutical products and pharmaceutical preparations (21); $\bullet$ Manufacture of computer, electronic and optical products (26); $\bullet$ Manufacture of air and spacecraft and related machinery (30.3)
- active enterprises with 3 and more employees engaged in innovation projects as a percentage of total enterprises 3 and more employees. The activities within their own Innovation projects: research and development activities carried out within the company, acquisition of research and development services, staff training on adopted and/or planned Innovations, technical and aesthetic design (design), acquisition of licences and patents, acquisition or development of software, databases and data analysis services, acquisition of computer hardware, network and telecommunications equipment, acquisition of machinery, equipment and facilities for adopted or planned Innovations, marketing for the launch of new goods and/or services, other activity.
- active enterprises with 3 and more employees using digital platforms as a percentage of total enterprises with 3 and more employees
- number of online services made available to citizens by the local public administration. The priority areas of the eGovernment according to the European Action Plan 2016-2020, are based on the following specific indicators:
  - User Centricity: indicates the extent to which a service is provided online, its mobile friendliness and usability of the service (in terms of available online support and feedback mechanisms).
  - Transparency: indicates the extent to which governments are transparent about (i) the process of service delivery, (ii) the responsibilities and performance of public organisations and (iii) the personal data processed in public services.
  - Cross-Border Mobility: indicates the extent to which users of public services from another European country can use the online services.
  - Key Enablers: indicates the extent to which technical and organisational preconditions for eGovernment service provision are in place, such as electronic identification and authentic sources.

*Pillar X - Business Sophistication*

The indicators selected for the analysis are:

- business fragmentation. Percentage share of micro, small and medium enterprises in total (reversed). In official statistics SMEs are identified as enterprises with fewer than 250 persons employed. This is a big category and encompasses enterprises with different ownership structures and varying numbers of employees and levels of economic activity. In the non-financial business economy (NACE Rev.2 Sections B to J and L to N and Division S95) enterprises employing fewer than 250 persons make up over 99.9% of all enterprises and they account for around four-fifths of total employment in the contribute about 68.8% of the total turnover. But SMEs show a low level of internalization compared to large companies in the same sector. In an increasingly globalized world, the high incidence of small and medium-sized enterprises represents an element of weakness to face competition in international markets. This explains the reverse relationship with attractiveness.
- agriculture forestry and fishing specialization index - value added^6^ (reversed). It is measured by the ratio between the share of agriculture in overall economy of the territory and its share overall Italian economy. It has a reverse relationship with attractiveness.
- industry specialization index - value added. It is measured by the ratio between the share of industry in overall economy of the territory and its share overall Italian economy. In addition to manufacturing industries, this sector also includes mining and quarrying and the supply of electricity, gas and water. The top 10 list of provinces most specialised in industry is dominated by the Northern provinces (Vicenza, Pordenone, Lecco, Belluno, Treviso etc.). Industrial activities contributes more in terms of value added than employment to the overall non-financial business economy, indicating an above average labour productivity.^7^ This explains the positive relationship with attractiveness.
- construction specialization index - value added. It is measured by the ratio between the share of construction in overall economy of the territory and its share overall Italian economy. Construction covers the development of building projects and construction of residential and non-residential buildings, as well as civil engineering of roads, railroads, utility projects, etc., and specialised construction activities. Contrary to industry, the construction sector reported relatively low levels of apparent labour productivity. This explains the reverse relationship with attractiveness.
- services specialization index - value added. It is measured by the ratio between the share of services in overall economy of the territory and its share overall Italian economy. This category covers a wide range of economic activities, including wholesale and retail trade, repair of motor vehicles and household goods; hotels and restaurants; transport and communication. The NUTS3 regions in Italy most specialised within these economic activities are all well-known tourist destination located in the South (Agrigento, Reggio Calabria, Barletta-Andria-Trani, Napoli). Data show that where the share of trade (wholesale and retail) in services is high, the manufacturing vocation of the territory is low: in the Southern provinces the percentage incidence of trade is two out of three in terms of employment and at the same time the share of industry is 9% compared to 45% of the most industrialized provinces (Vicenza, Pordenone etc.). This empirical evidence explains the reverse relationship with attractiveness, being the trade a labor-intensive sector and therefore with low production efficiency.
- entrepreneurship intensity. Number of active enterprises per thousand inhabitants. It is an indicator of the liveliness of the local economic system.
- number of total businesses registered in the cultural production system divided on the total provincial economy

*Pillar XI - Innovation*

The indicators selected for the analysis are:

- propensity to patent. total number of patent applications to the european patent office (epo). Values per million inhabitants. Patents^8^ are granted for inventions which are novel, inventive (non-obvious) and have an industrial application (useful) and he patent holder is in a position to set a higher-than-competitive price for the corresponding good or service, which allows recovery of Innovation costs. Patents are territorial rights: the invention is protected only in those countries in which these patents have been granted.
- propensity to patent - number of patents applications to the italian patent office (uibm). Values per million inhabitants. The European Patent Convention, signed in Munich on October 5, 1973, allows every citizen or resident of a Member State to make use of a single European procedure for the granting of patents, based on a homogeneous body of fundamental patent laws.
- registered patents to the italian patent office (uibm) -values per million inhabitants. Italy is a member of the European Patent Organization (EPO).
- registered trademarks by province of registration in italy. Values per million inhabitants. The brand is a distinctive sign. It is used to distinguish the products or services of a company from those offered on the market by other companies in the sector.
- brain gain/drain or mobility of italian graduates. Net migration rate of Italian graduates 25-39 years per 1000 inhabitants per 1000 resident graduates. It is calculated as the ratio between the migratory balance, enrolled in the registry - deleted from the registry for transfer of residence, of graduates aged 25-29 and residents with tertiary education (degree, AFAM, PhD) of the same age group, including interregional and interprovincial movements. Positive numbers mean a territory has had more in-migration than out-migration “brain gain”, while negative numbers mean the opposite “brain drain”. Therefore, the most serious risk for an unattractive territory is “desertification”, not only in the productive sense but also in the demographic sense.
- cultural business employees as a percentage of total active business employees. See NACE classification for the codes of the component sectors.

**Table 13:** Pillars by province

Region	Province	Pillar I	Pillar II	Pillar III	Pillar IV	Pillar V	Pillar VI	Pillar VII	Pillar VIII	Pillar IX	Pillar X	Pillar XI
Piemonte	Torino	0.76	0.35	0.66	0.04	0.49	0.72	0.76	0.38	0.42	0.49	1.35
Piemonte	Vercelli	0.67	− 0.30	− 0.66	− 0.08	0.35	0.06	0.21	− 0.54	− 0.42	0.29	− 0.69
Piemonte	Novara	0.61	0.02	− 0.26	− 0.63	0.51	0.03	0.83	− 0.25	− 0.08	0.57	0.12
Piemonte	Cuneo	0.86	− 0.40	− 0.36	0.26	1.16	− 0.14	0.54	− 0.05	− 0.32	0.08	− 0.16
Piemonte	Asti	0.70	− 0.01	− 0.41	0.09	0.92	− 0.13	0.24	− 0.29	− 0.46	− 0.15	− 0.45
Piemonte	Alessandria	0.54	− 0.43	− 0.38	− 1.20	0.09	0.29	0.33	− 0.44	− 0.39	0.10	− 0.45
Piemonte	Biella	0.75	− 0.18	− 0.62	− 0.69	0.35	0.57	0.66	− 0.46	− 0.65	0.50	− 0.65
Piemonte	Verbano C.O.	0.80	− 0.87	− 0.49	− 0.64	0.83	− 0.24	0.28	− 0.42	− 0.99	− 0.36	− 0.51
Valle d’Aosta	Aosta	1.04	0.65	− 0.87	− 0.37	0.93	− 0.55	0.41	− 0.12	− 0.10	− 0.20	− 0.41
Liguria	Imperia	0.12	0.21	− 0.51	0.03	0.04	− 0.46	− 0.37	− 0.63	− 0.98	− 0.94	− 0.58
Liguria	Savona	0.45	− 0.11	− 0.02	− 0.01	0.16	0.78	0.18	− 0.57	− 0.32	− 0.18	− 0.38
Liguria	Genova	0.39	− 0.51	0.59	− 0.08	0.21	0.64	0.38	0.08	0.03	− 0.05	− 0.24
Liguria	La Spezia	0.34	− 0.63	0.18	− 0.48	0.00	0.32	0.07	− 0.28	− 0.20	− 0.19	− 0.42
Lombardia	Varese	0.45	0.03	− 0.38	0.88	0.88	− 0.06	0.69	− 0.15	0.29	0.61	− 0.02
Lombardia	Como	0.50	− 0.01	− 0.46	0.86	1.24	− 0.17	0.48	− 0.27	− 0.11	0.39	0.09
Lombardia	Sondrio	0.43	0.17	− 1.54	− 0.49	1.59	0.19	0.43	− 0.35	− 0.42	− 0.29	− 0.70
Lombardia	Milano	0.66	0.38	2.42	0.53	0.69	0.83	1.32	2.56	1.68	1.09	3.14
Lombardia	Bergamo	0.45	0.01	− 0.35	0.18	1.14	0.25	0.37	0.11	− 0.02	0.52	− 0.23
Lombardia	Brescia	0.49	− 0.34	− 0.12	0.67	1.22	− 0.25	0.51	0.18	− 0.28	0.61	− 0.10
Lombardia	Pavia	0.53	0.19	− 0.12	− 0.31	0.62	0.42	0.97	− 0.28	− 0.25	0.20	− 0.49
Lombardia	Cremona	0.58	− 0.01	− 0.41	− 0.11	0.91	− 0.19	0.69	− 0.30	− 0.28	0.54	− 0.34
Lombardia	Mantova	0.66	− 0.72	− 0.21	− 0.38	0.83	− 0.67	0.51	− 0.25	− 0.74	0.52	− 0.28
Lombardia	Lecco	0.64	0.40	− 0.56	0.97	1.42	0.35	0.67	− 0.44	− 0.43	0.87	− 0.01
Lombardia	Lodi	0.56	0.27	− 0.34	− 0.24	0.73	− 0.08	0.51	− 0.67	− 0.48	0.42	− 0.65
Lombardia	Monza Brianza	0.51	0.48	− 0.31	0.56	1.21	0.28	0.78	0.12	0.44	0.66	0.04
Trentino Alto Adige	Bolzano	0.72	0.37	− 0.72	0.74	0.27	− 0.34	1.06	0.70	0.68	− 0.01	− 0.12
Trentino Alto Adige	Trento	0.96	1.06	− 0.54	0.78	1.32	0.82	0.47	0.03	0.97	0.12	0.01
Veneto	Verona	0.44	− 0.15	− 0.01	0.53	1.08	0.19	0.48	0.21	0.03	0.46	0.19
Veneto	Vicenza	0.14	0.14	− 0.11	0.76	1.41	0.08	0.62	0.07	0.24	1.23	0.23
Veneto	Belluno	0.29	− 0.13	− 0.52	0.73	1.44	− 0.08	0.69	− 0.13	− 0.24	0.44	− 0.54
Veneto	Treviso	0.53	0.10	− 0.04	0.98	1.47	0.31	0.54	− 0.11	0.04	0.65	0.11
Veneto	Venezia	0.58	0.12	1.53	0.56	0.76	0.50	0.38	0.16	− 0.01	− 0.02	− 0.09
Veneto	Padova	0.42	− 0.32	0.12	0.80	1.39	0.68	0.41	0.31	0.22	0.57	0.26
Veneto	Rovigo	0.42	− 0.44	− 0.36	− 0.03	1.04	0.63	0.17	− 0.54	− 0.82	− 0.08	− 0.75
Friuli Venezia Giulia	Udine	1.14	− 0.33	− 0.26	0.62	1.17	0.91	0.43	− 0.10	− 0.39	0.24	0.43
Friuli Venezia Giulia	Gorizia	0.89	− 0.66	0.10	0.02	0.47	− 0.76	0.30	− 0.33	0.23	0.41	− 0.50
Friuli Venezia Giulia	Trieste	0.93	− 0.27	0.71	− 0.15	0.50	1.49	1.07	− 0.21	1.18	0.27	0.03
Friuli Venezia Giulia	Pordenone	1.00	− 0.88	0.00	0.45	1.06	0.00	0.41	− 0.12	0.29	0.59	0.35
Emilia Romagna	Piacenza	0.55	− 0.13	− 0.38	− 0.63	0.62	1.01	0.51	− 0.39	− 0.07	0.47	− 0.31
Emilia Romagna	Parma	0.43	− 0.81	− 0.07	0.15	0.60	1.36	0.72	0.04	0.07	0.72	0.51
Emilia Romagna	Reggio Emilia	0.84	0.45	− 0.08	0.39	0.56	0.22	0.33	− 0.15	− 0.31	0.91	0.18
Emilia Romagna	Modena	0.59	− 0.10	− 0.37	0.37	0.48	0.64	0.76	0.04	0.42	0.90	0.75
Emilia Romagna	Bologna	0.89	0.34	0.30	0.80	0.61	1.41	0.87	0.20	0.63	0.77	1.37
Emilia Romagna	Ferrara	0.91	− 0.77	− 0.35	− 0.33	− 0.02	1.15	0.52	− 0.39	− 0.13	− 0.09	0.07
Emilia Romagna	Ravenna	0.86	− 0.02	− 0.15	0.59	0.91	− 0.09	0.43	− 0.12	0.13	0.14	0.07
Emilia Romagna	Forli Cesena	0.69	− 0.21	− 0.51	1.01	0.90	0.28	0.25	− 0.24	− 0.11	0.11	− 0.03
Emilia Romagna	Rimini	0.69	− 1.17	0.06	0.74	0.78	− 0.30	− 0.15	− 0.37	0.83	0.18	− 0.09
Marche	Pesaro Urbino	0.25	− 1.07	− 0.43	0.48	0.20	0.46	− 0.03	− 0.48	− 0.04	0.36	− 0.35
Marche	Ancona	0.46	− 0.02	− 0.01	0.27	0.33	0.25	0.37	− 0.31	0.48	0.46	0.23
Marche	Macerata	0.00	0.28	− 0.54	0.47	0.46	0.28	− 0.08	− 0.46	− 0.63	0.19	− 0.30
Marche	Ascoli Piceno	0.06	0.20	− 0.45	0.22	0.25	0.98	− 0.32	− 0.68	− 0.16	0.26	− 0.33
Marche	Fermo	0.03	− 0.25	− 0.50	0.20	0.40	0.32	0.00	− 0.74	− 0.30	0.53	− 0.42
Toscana	Massa Carrara	0.29	− 0.57	− 0.44	− 0.06	− 0.47	0.06	0.07	− 0.32	− 0.53	− 0.16	− 0.42
Toscana	Lucca	0.35	− 0.73	− 0.39	0.51	− 0.18	0.04	0.07	− 0.34	− 0.41	0.27	− 0.20
Toscana	Pistoia	0.45	− 0.13	− 0.22	0.54	0.33	0.10	− 0.12	− 0.60	− 0.23	− 0.45	− 0.35
Toscana	Firenze	0.76	0.52	0.84	0.86	− 0.37	1.16	0.68	0.12	0.50	0.62	0.54
Toscana	Livorno	0.69	0.71	− 1.30	0.25	− 0.41	− 0.14	− 0.20	− 0.22	0.04	0.01	− 0.44
Toscana	Pisa	0.57	0.33	− 0.10	0.72	− 0.15	0.95	0.56	− 0.26	− 0.06	0.19	0.39
Toscana	Arezzo	0.66	− 0.16	− 0.50	0.67	0.23	− 0.30	− 0.03	− 0.37	− 0.49	0.50	− 0.35
Toscana	Siena	0.66	0.92	− 0.38	0.02	0.33	0.38	0.35	− 0.18	0.49	− 0.08	− 0.20
Toscana	Grosseto	0.32	0.24	− 0.90	0.35	− 0.23	− 0.38	− 0.06	− 0.52	− 0.76	− 0.86	− 0.56
Toscana	Prato	0.75	0.72	− 0.15	0.91	− 0.04	0.33	0.33	− 0.26	− 0.73	0.75	− 0.43
Umbria	Perugia	0.03	− 0.42	− 0.58	0.88	0.19	0.56	0.13	− 0.60	− 0.27	− 0.06	− 0.37
Umbria	Terni	− 0.14	− 0.85	− 0.51	− 0.17	− 0.03	0.08	− 0.01	− 0.67	− 0.55	− 0.19	− 0.57
Lazio	Viterbo	− 0.59	− 0.11	− 0.50	− 1.53	− 0.27	0.05	− 0.28	− 0.62	− 0.74	− 0.94	− 0.58
Lazio	Rieti	− 0.65	− 0.93	− 0.55	− 0.11	− 0.54	0.24	− 0.59	− 0.85	− 1.63	− 0.94	− 0.54
Lazio	Roma	− 0.67	− 0.57	1.08	0.18	− 0.59	0.42	0.77	0.97	0.45	0.09	1.04
Lazio	Latina	− 0.98	− 0.31	− 0.56	− 0.23	− 0.51	− 0.03	− 0.43	− 0.50	− 0.20	− 0.28	− 0.78
Lazio	Frosinone	− 0.89	− 0.70	− 0.40	− 0.46	− 0.51	− 0.38	− 0.57	− 0.52	− 1.20	0.09	− 0.75
Campania	Caserta	− 1.11	− 0.42	− 0.29	− 1.63	− 1.24	− 0.92	− 1.12	− 0.58	− 0.91	− 0.99	− 0.82
Campania	Benevento	− 0.86	− 2.18	− 0.50	− 1.30	− 0.60	0.00	− 0.91	− 0.76	− 0.13	− 0.97	− 0.81
Campania	Napoli	− 0.84	1.05	− 0.06	− 1.31	− 1.15	− 1.23	− 0.98	0.04	0.19	− 0.58	− 0.68
Campania	Avellino	− 0.99	− 0.42	− 0.29	− 0.47	− 0.73	0.05	− 0.62	− 0.54	− 0.80	− 0.50	− 0.87
Campania	Salerno	− 1.11	− 1.21	− 0.59	− 0.83	− 0.90	− 0.54	− 1.03	− 0.45	− 0.18	− 0.68	− 0.71
Abruzzo	L’Aquila	− 0.61	0.31	− 0.37	− 0.44	− 0.84	0.94	− 0.35	− 0.53	− 0.94	− 0.76	− 0.58
Abruzzo	Teramo	− 0.82	− 1.31	− 0.39	− 0.58	− 0.42	0.35	− 0.37	− 0.55	− 0.78	− 0.23	− 0.61
Abruzzo	Pescara	− 0.76	− 1.68	− 0.36	0.17	− 0.21	0.39	− 0.28	− 0.69	0.05	− 0.26	− 0.37
Abruzzo	Chieti	− 0.79	− 1.17	− 0.49	0.26	− 0.19	0.27	− 0.66	− 0.24	− 0.74	0.16	− 0.82
Molise	Campobasso	− 0.37	0.28	− 0.73	− 1.07	− 0.17	− 0.20	− 0.58	− 0.83	− 1.01	− 0.73	− 0.75
Molise	Isernia	− 0.64	0.63	− 0.65	− 1.05	− 0.69	0.65	− 0.54	− 0.62	− 1.33	− 0.85	− 0.82
Puglia	Foggia	− 1.75	− 0.42	− 0.56	− 0.52	− 0.93	− 0.99	− 1.30	− 0.50	− 0.59	− 1.17	− 1.10
Puglia	Bari	− 1.46	0.49	0.03	0.56	− 0.32	− 0.36	− 0.64	− 0.10	0.30	− 0.45	− 0.55
Puglia	Taranto	− 1.32	0.22	− 0.14	− 0.19	− 0.68	− 1.06	− 1.10	− 0.42	− 0.01	− 0.52	− 1.11
Puglia	Brindisi	− 1.08	− 0.25	− 0.34	0.30	− 0.64	− 1.37	− 0.61	− 0.48	− 0.62	− 0.73	− 1.02
Puglia	Lecce	− 1.19	− 0.74	− 1.12	− 0.02	− 0.20	− 0.44	− 1.19	− 0.46	− 0.35	− 0.81	− 0.77
Puglia	Barletta Andria Trani	− 1.42	− 0.33	− 1.19	− 0.61	− 0.35	− 1.15	− 1.30	− 0.36	− 1.29	− 0.80	− 0.95
Basilicata	Potenza	− 0.70	0.08	− 0.88	− 0.82	− 0.54	− 0.24	− 1.36	− 0.26	− 0.16	0.07	− 1.00
Basilicata	Matera	− 0.75	− 0.47	− 0.89	− 0.55	− 0.26	− 0.28	− 0.68	− 0.51	− 0.20	− 1.01	− 0.93
Calabria	Cosenza	− 0.59	− 0.33	− 0.74	− 1.24	− 1.44	− 0.47	− 1.30	− 0.75	− 0.33	− 1.07	− 0.89
Calabria	Catanzaro	− 0.79	− 1.39	− 0.56	− 0.80	− 0.85	− 0.20	− 0.76	− 0.32	0.02	− 1.03	− 0.92
Calabria	Reggio Calabria	− 0.55	0.35	− 0.61	− 1.22	− 1.38	− 0.85	− 1.61	− 0.65	− 0.38	− 1.16	− 1.12
Calabria	Crotone	− 0.58	− 0.99	− 1.34	− 0.67	− 1.66	− 1.45	− 1.25	− 1.02	− 0.44	− 1.14	− 1.32
Calabria	Vibo Valentia	− 0.95	− 0.38	− 0.58	− 0.04	− 1.51	− 1.08	− 1.27	− 0.66	0.07	− 1.18	− 1.03
Sicilia	Trapani	− 0.44	1.14	− 0.51	− 1.57	− 1.30	− 1.25	− 1.21	− 0.83	− 0.76	− 1.04	− 0.98
Sicilia	Palermo	− 0.52	0.64	− 0.18	− 0.58	− 1.36	− 1.00	− 0.94	− 0.54	− 0.27	− 0.81	− 0.56
Sicilia	Messina	− 0.91	0.27	− 0.57	− 1.18	− 0.96	− 0.64	− 1.40	− 0.69	− 0.50	− 0.74	− 0.92
Sicilia	Agrigento	− 0.59	0.50	− 0.83	− 1.01	− 1.62	− 0.91	− 1.23	− 0.91	− 1.46	− 1.23	− 1.07
Sicilia	Caltanissetta	− 0.54	− 0.18	− 0.72	− 0.90	− 1.29	− 1.70	− 1.53	− 1.31	− 1.00	− 1.44	− 1.30
Sicilia	Enna	− 0.59	0.20	− 0.61	0.49	− 2.04	− 1.09	− 1.30	− 0.96	− 1.24	− 1.15	− 1.21
Sicilia	Catania	− 0.94	0.26	− 0.14	− 0.38	− 1.18	− 1.41	− 0.92	− 0.65	− 0.09	− 0.82	− 0.85
Sicilia	Ragusa	− 0.86	0.48	− 0.87	− 0.32	− 0.66	− 1.36	− 1.18	− 0.79	− 0.10	− 1.05	− 0.84
Sicilia	Siracusa	− 0.90	− 0.04	− 0.60	− 0.71	− 1.34	− 1.73	− 1.17	− 0.61	− 0.52	− 0.11	− 0.97
Sardegna	Sassari	− 0.22	0.90	− 0.61	− 0.20	− 1.49	− 0.88	− 0.39	− 0.46	− 0.73	− 0.87	− 0.70
Sardegna	Nuoro	− 0.40	1.23	− 1.16	− 0.65	− 1.47	− 0.85	− 0.68	− 0.68	− 0.86	− 1.21	− 0.93
Sardegna	Cagliari	− 0.33	2.74	− 0.45	0.51	− 1.27	0.24	0.11	− 0.18	− 0.48	0.10	− 0.51
Sardegna	Oristano	− 0.45	1.60	− 0.92	0.36	− 1.63	0.10	− 0.70	− 0.63	− 1.30	− 1.11	− 0.72

## Footnotes

1 Among the economists, the fiercest opponent to the concept of competitiveness of a country is Paul Krugman (Krugman 1994) who defines country competitiveness as a dangerous obsession with politicians when they claim to put it at the top of their priority agenda. The main argument of the MIT professor is that competitiveness is in itself an empty word and acquires its meaning only by referring to productivity (“... a poetic way of saying productivity ... ”). In fact, the most commonly used single indicator of competitiveness at the country level is the labour cost per unit of product (ULC) calculated as the ratio between unit labour cost (per worker or per hour worked) and labor productivity (added value for worker or per hour worked). If productivity is certainly to be considered as a key factor of a country’s competitiveness, the link between competitiveness and well-being is a mutual one. Empirical evidence highlights the virtuous circle between productivity-competitiveness-income per capita, considering that the most competitive countries in international rankings are also those characterized by a higher standard of living measured by per capita income.

2 I.stat (Istat), Istat (Bes), Istat (Indicatori territoriali per le Politiche di sviluppo), Istat (A misura di comune), Istat (Companies Permanent Census), Istat (ASIA), Istat (COEWEB), Istat (BES), Minister of Justice (DG-STAT), Fondazione Etica su dati Amministrazione Trasparente, OECD (PISA), Minister of Economic Development, UIBM database.
